# A world checklist of Onychophora (velvet worms), with notes on nomenclature and status of names

**DOI:** 10.3897/zookeys.211.3463

**Published:** 2012-07-25

**Authors:** Ivo de Sena Oliveira, V. Morley St. J. Read, Georg Mayer

**Affiliations:** 1 Animal Evolution and Development, Institute of Biology, University of Leipzig, Talstraße 33, D-04103 Leipzig, Germany; 2 Museo de Zoologia QCAZ, Escuela de Ciencias Biológicas, Pontificia Universidad Católica del Ecuador, Av. 12 de Octubre 1076 y Roca, 17-01-2184 Quito, Ecuador

**Keywords:** Peripatidae, Peripatopsidae, species list, catalogue, taxonomy

## Abstract

Currently, the number of valid species of Onychophora is uncertain. To facilitate taxonomic work on this understudied animal group, we present an updated checklist for the two extant onychophoran subgroups, Peripatidae and Peripatopsidae, along with an assessment of the status of each species. According to our study, 82 species of Peripatidae and 115 species of Peripatopsidae have been described thus far. However, among these 197 species, 20 are *nomina dubia* due to major taxonomic inconsistencies. Apart from *nomina dubia*, many of the valid species also require revision, in particular representatives of *Paraperipatus* within the Peripatopsidae, and nearly all species of Peripatidae. In addition to extant representatives, the record of unambiguous fossils includes three species with uncertain relationship to the extant taxa. For all species, we provide a list of synonyms, information on types and type localities, as well as remarks on taxonomic and nomenclatural problems and misspellings. According to recent evidence of high endemism and cryptic speciation among the Peripatidae and Peripatopsidae, previous synonyms are revised. Putative mutations, subspecies and variations are either raised to the species status or synonymised with corresponding taxa. In our revised checklist, we follow the rules and recommendations of the International Code of Zoological Nomenclature to clarify previous inconsistencies.

## Introduction

**Figure 1.**
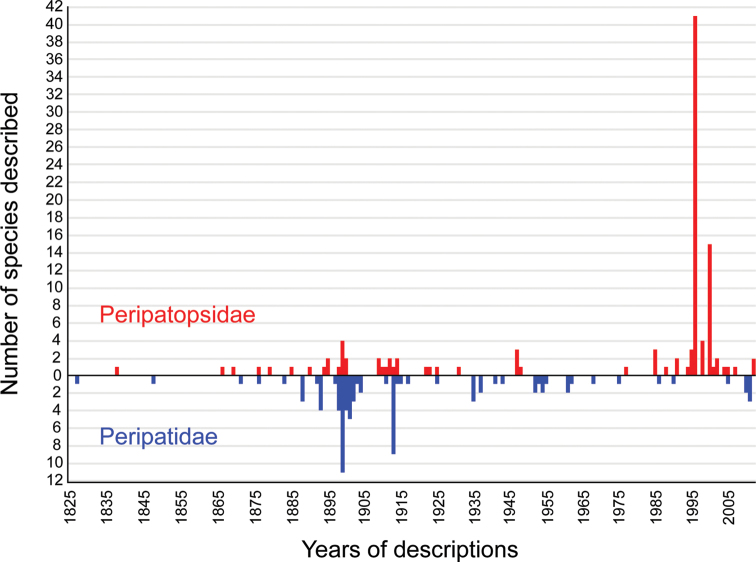
Diagram illustrating the number of species descriptions of Onychophora per year since the very first description of an onychophoran species by Guilding (1826). Note the numerous gaps in the taxonomical work.

Although onychophorans play an important role in studies of animal evolution, biogeography and conservation (Sedgwick 1908; Brues 1923; Brinck 1957; Ruhberg 1985; Monge-Nájera 1995; New 1995; Reid 1996), the taxonomy and species diversity of this taxon are understudied. According to a recent estimate, a total of 180 extant onychophoran species have been described since the first description in 1826, including 73 species of Peripatidae and 107 species of Peripatopsidae (Fig. 1; Mayer and Oliveira 2011). However, the validity of many of these species is uncertain. Although a revision at the species level seems timely, it represents a challenge because several original descriptions and revisions are difficult to access or they have been published in different languages, including Latin (Guilding 1826), Russian (Sänger 1871), Dutch (Weber 1898), French (Bouvier 1905), Italian (Clark 1913a), Spanish (Scorza 1953), German (Ruhberg 1985), Portuguese (Oliveira and Wieloch 2005) and English (e.g., Reid 1996; Oliveira et al. 2011).

In this study, we compiled a checklist of all described species of Onychophora and re-evaluated their species status following the International Code of Zoological Nomenclature (ICZN). Our checklist summarizes information on valid species names, synonyms, description language, type locality, type designation and location of holotypes. Additional remarks highlight aspects relevant for future work, which will help improve our knowledge of the taxonomy and species diversity of Onychophora.

## Methods

The checklist of all described species of Onychophora was compiled by gathering, translating and interpreting information from the literature and from museums’ databases (Natural History Museum of London – NHM, United Kingdom, and Muséum National d’Histoire Naturelle of Paris, France). Information on cryptic speciation and point endemism, common among species of Peripatidae and Peripatopsidae (Trewick 1998; Daniels et al. 2009; Oliveira et al. 2011), as well as precise collecting data were used to assess the validity status of species. Previous synonyms were critically evaluated and ambiguous synonyms reconsidered. Putative mutations and variations, based on characters known to be intra-specifically variable, were synonymised, if they were reported from the species type locality. Putative subspecies and variations with precise collecting data, which occur far from the type locality (at least 30 km), were raised to the species status. This approach is justifiable, given high cryptic diversity and endemism among onychophorans, including representatives of both Peripatidae and Peripatopsidae. The data available suggest that most clades, which are regarded as species or cryptic species, are found at localities lying at least 10–30 km apart (Reid 1996; Lacorte et al. 2011; Oliveira et al. 2011). We used this information as a guideline for assessing the validity of the species, i.e., if two putative clades occur at a distance of over 30 km from each other, they are likely to be separate species.

**Figure 2.**
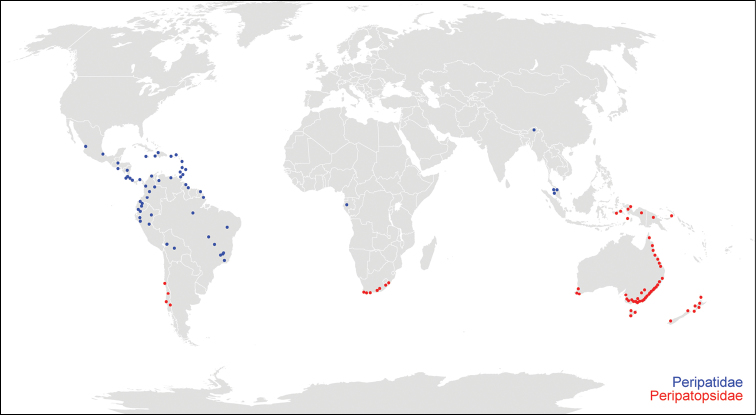
Overview map with type localities of valid onychophoran species worldwide. The type localities of the Peripatidae species are indicated by blue dots, those of the Peripatopsidae species by red dots.

**Figure 3.**
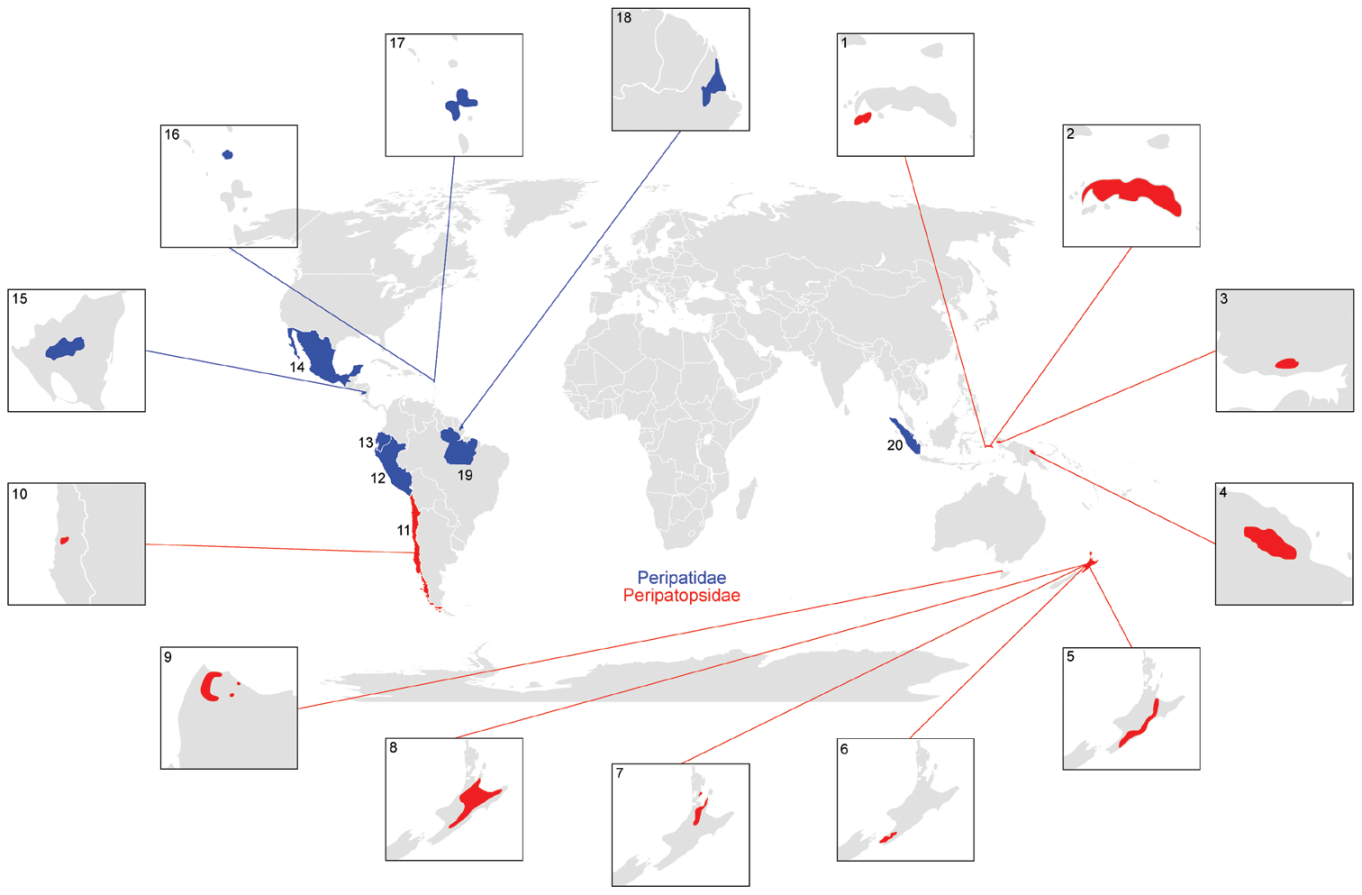
Overview map withlocalities of onychophoran species regarded as *nomina dubia* in the present work. Note that the localities of these species are imprecise. The localities of the Peripatidae species are indicated in blue, those of the Peripatopsidae species in red. Numbers refer to the following species: **1** *Paraperipatus amboinensis* Pflugfelder, 1948 **2** *Paraperipatus stresemanni* Bouvier, 1914b **3** *Paraperipatus leopoldi* Leloup, 1931 **4** *Paraperipatus schultzei* Heymons, 1912**5** *Peripatoides morgani* Trewick, 1998 **6** *Peripatoides novaezealandiae* (Hutton, 1876) **7** *Peripatoides aurorbis* Trewick, 1998 **8** *Peripatoides sympatrica* Trewick, 1998 **9** *Ooperipatellus cryptus* Jackson & Taylor, 1994 **10** *Paropisthopatus costesi* (Gravier & Fage, 1925) **11** *Metaperipatus blainvillei* (Gervais, 1837) **12** *Oroperipatus peruanus* (Grube, 1876) **13** *Oroperipatus quitensis* (Schmarda, 1871) **14** *Oroperipatus goudoti* (Bouvier, 1899c) **15** *Epiperipatus nicaraguensis* (Bouvier, 1900a) **16** *Peripatus antiguensis* Bouvier, 1899c **17** *Peripatus bavaysi* Bouvier, 1899c **18** *Macroperipatus geayi* (Bouvier, 1899c) **19** *Epiperipatus tucupi* (Froehlich, 1968) **20** *Eoperipatus sumatranus* (Sedgwick, 1888).

In the list, valid species names are sorted in alphabetical order and numbered consecutively within Peripatidae and Peripatopsidae. Fossil species are numbered separately at the end of the list. *Nomina dubia* are left unnumbered and listed after the valid names of each genus. No abbreviations are used for genus names in order to avoid confusion among species with a similar epithet. Each taxon/species name is accompanied by the corresponding author and year. Synonyms are arranged in chronological order and only the first reference mentioning a particular synonym is cited. Misspellings were not regarded as synonyms but are discussed in the Remarks section for each species. Only the data on the holotype are considered since it represents a single specimen, while syntypes may originate from different localities and, thus, they might represent different species. Only the type locality data are provided for each species rather than its putative range of distribution. Old locality names were updated and country names are provided in capitals according to their current political borders. The International System of Units (SI) is used throughout the list. The original information found in the literature is provided along with the converted units (1ft = 0.3048 m; 1 mile = 1.6 km). All available data on the type localities of valid species (Fig. 2) as well as on the localities of species regarded as *nomina dubia* herein (Fig. 3) are included into a world map based on the information obtained using the freeware Google Earth®.

## Checklist

### ONYCHOPHORA Grube, 1853

#### I. PERIPATIDAE Evans, 1901a

Type genus: *Peripatus* Guilding, 1826

Remark: A thorough revision of the group, particularly neotropical genera, is required (Peck 1975: 343).

##### *Eoperipatus* Evans, 1901a

Type species: *Eoperipatus horsti* (Evans, 1901a), designated herein (see Remarks).

Remarks: So far, no type species has been designated for the genus. Taking into account the recommendations of the ICZN (Art. 67) and the amount of information available in the literature, we designate *Eoperipatus horsti* as the type species of the genus since it is the only originally included nominal species for which data from both sexes are available.

1. *Eoperipatus butleri* Evans, 1901b

Synonyms: None.

Holotype: Not designated.

Type locality: Malaysia, Perak, Bukit Larut (Larut Hills), 1,219 m (4,000 ft).

Language of species description: English.

Remarks: Bouvier (1905) did not recognise differences between *Eoperipatus butleri* and *Eoperipatus weldoni* and suggested they are synonyms, although their type localities lie over 300 km apart. Based on differences described by Evans (1901a, 1901b), we regard *Eoperipatus butleri* and *Eoperipatus weldoni* as separate species. A thorough revision of both species is required.

2. *Eoperipatus horsti* Evans, 1901a

Synonyms: None.

Holotype: Not designated.

Type locality: Malaysia, Kelantan, Kuala Aring.

Language of species description: English.

Remarks: Kloss (1926) recorded a specimen from sea level, suggesting that it is a lowland species. Van der Lande and Holthuis (1986: 18) discuss the possibility that the species is a variation of *Eoperipatus sumatranus*, and later, all the Malaysian species were treated under the name *Eoperipatus sumatranus* (van der Lande 1988: 13). We regard *Eoperipatus horsti* as a separate species and the latter as a *nomen dubium* (see Remarks for *Eoperipatus sumatranus* below). Requires revision.

3. *Eoperipatus weldoni* Evans, 1901a

Synonyms: None.

Holotype: Not designated.

Type locality: Malaysia, Bukit Besar, “on the boundary line between the States of Nawng Chick and Jalor, a full day’s journey from the town of Patani”, 686 m (2,250 ft) (see Remarks).

Language of species description: English.

Remarks: The name Bukit Besar is currently attributed to different localities in Malaysia but evidence indicates that the putative type locality might be within the limits of the Bukit Perangin Forest Reserve, northern Malaysia, next to the border with the southern part of Thailand. An area referred to as Bukit Besar, located in the western part of the Forest Reserve, fits with the information on the altitude (690 m) and the distance from Patani (104 km from Pattani, South Thailand) provided by Evans (1901a). Moreover, the Bukit Perangin Forest Reserve is situated next to the border of the province Yala (also known as Jala or Jolor), which might have been spelt as Jalor by the author. The current name and position of the state of Nawng Chick could not be found. Kloss (1926) stated that in contrast to *Eoperipatus horsti*, *Eoperipatus weldoni* is found in mountainous habitats. Van der Lande and Holthuis (1986: 18) discuss the possibility that the species is a variation of *Eoperipatus sumatranus* (van der Lande and Holthuis 1986: 18), and later, all Malaysian species were treated under the name *Eoperipatus sumatranus* (van der Lande 1988: 13), which we regard as a *nomen dubium* (see Remarks for *Eoperipatus sumatranus*). Thus, *Eoperipatus weldoni* is most likely a separate species, although it requires revision.

***Nomen dubium***

*Eoperipatus sumatranus* (Sedgwick, 1888)

Synonyms: *Peripatus sumatranus*, by original designation (Sedgwick 1888: 485); *Eoperipatus sumatranus* (Evans 1901a: 484).

Holotype: Not designated (see Remarks).

Type locality: Unknown. Van der Lande and Holthuis (1986: 19) assumed it might be Mount Arjuno in East Java rather than a locality in Sumatra. However, apart from the doubtful record of *Eoperipatus sumatranus*, no onychophorans have been reported from Java or Sumatra thus far (see Remarks).

Language of species description: English.

Remarks: Holotype not clearly designated in the original description. The type locality of this species is unlikely to be Sumatra since the collector might have never been on this island (van der Lande and Holthuis 1986). The suggestion of Mount Arjuno in East Java as a putative type locality (van der Lande and Holthuis 1986) is in our view a mere guess. Therefore, we regard *Eoperipatus sumatranus* as a *nomen dubium* since neither the holotype nor the type locality is known, which makes a revision difficult.

##### *Epiperipatus* (Clark, 1913b)

Type species: *Epiperipatus edwardsii* (Blanchard, 1847), by original designation (Clark 1913b: 17).

Remarks: The name was introduced initially as a subgenus of *Peripatus* (see Clark 1913b). It has been commonly used since then as a genus name, although it has never been raised formally to this status (Read 1988a: 189). The monophyly of *Epiperipatus* is uncertain and the entire genus requires revision (Peck 1975; Oliveira et al. 2010).

4. *Epiperipatus acacioi* (Marcus & Marcus, 1955)

Synonyms: *Peripatus ouropretanus* (junior synonym; Trindade 1958: 520; see Remarks); *Peripatus acacioi*, by original designation (Marcus and Marcus 1955: 189); *Peripatus (Macroperipatus) acacioi* (Froehlich 1968: 168; see Remarks); *Macroperipatus acacioi* (Peck 1975: 346); *Epiperipatus acacioi* (Oliveira et al. 2010: 21).

Holotype: Not designated (see Remarks).

Type locality: Brazil, Minas Gerais, Ouro Preto, Ecological Station of Tripuí, 20°23'45"S, 43°34'33"W, 1,215 m.

Language of species description: English.

Remarks: The name *Peripatus ouropretanus* is regarded here as a *nomen nudum*. The species has been misspelt as *Penipatus* [sic] (*Macroperipatus acacioi*) and *Penipatus* [sic] (*Macroperipatus*) *acacioi* in Vasconcellos et al. (2004: 140). Specimens referred to as holotype and paratype by Sampaio-Costa et al. (2009: 557) and Oliveira et al. (2010: 21)are in fact syntypes according to the original description.

5. *Epiperipatus adenocryptus* Oliveira, Lacorte, Fonseca, Wieloch & Mayer,2011

Synonyms: *Epiperipatus analogos* (*nomen nudum*; Lacorte et al. 2010: 342; see Remarks).

Holotype: Deposited in the Instituto de Ciências Biológicas da Universidade Federal de Minas Gerais, Belo Horizonte, Brazil.

Type locality: Brazil, Minas Gerais, Santa Bárbara do Leste, Córrego dos Ferreiras, 19°58'59"S, 42°06'46"W, 1,050 m.

Language of species description: English.

Remarks: The name *Epiperipatus analogos* (see Lacorte et al. 2010: 342) is invalid according to the ICZN since it was used without providing a formal description (Art. 13) and depositing type specimens (Art. 16). Moreover, according to the ICZN (Art. 9.9), the proposition of this name in a proceedings abstract cannot be regarded as a valid publication for purposes of zoological nomenclature. Morphologically, *Epiperipatus adenocryptus* is very similar to *Epiperipatus paurognostus*. The species diagnosis contains morphological and molecular characters (Oliveira et al. 2011).

6. *Epiperipatus barbadensis* (Froehlich, 1962)

Synonyms: *Peripatus (Peripatus) dominicae barbadensis*, by original designation (Froehlich 1962: 325); *Peripatus dominicae barbadensis* (Peck 1975: 348); *Epiperipatus barbadensis* (Read 1988b: 237).

Holotype: Deposited in the Zoology Department of the Universidade de São Paulo, São Paulo, Brazil.

Type locality: Barbados Island, St. John, Codrington College.

Language of species description: English.

Remarks: See Read (1988b) for further details.

7. *Epiperipatus barbouri* (Brues, 1911)

Synonyms: *Peripatus barbouri*, by original designation (Brues 1911: 305); *Epiperipatus barbouri* (Peck 1975: 345).

Holotype: Deposited in the Museum of Comparative Zoology at Harvard University, Cambridge, USA.

Type locality: Grenada Island, Grand Etang, 548 m (1,800 ft).

Language of species description: English.

Remarks: See Read (1988b) for further details.

8. *Epiperipatus betheli* (Cockerell, 1913a)

Synonyms: *Peripatus (Epiperipatus) biolleyi* var. *betheli*, by original designation (Cockerell 1913a: 87).

Holotype: Not designated.

Type locality: Guatemala, Puerto Barrios.

Language of species description: English.

Remarks: We regard *Epiperipatus betheli* as a species rather than a variation of *Epiperipatus biolleyi* because of the great distance (808 km) between the type localities of the two species. Revision is required as it might reveal morphological and molecular differences between *Epiperipatus betheli* and *Epiperipatus biolleyi*. Requires revision.

9. *Epiperipatus biolleyi* (Bouvier, 1902a)

Synonyms: *Peripatus biolleyi*, by original designation (Bouvier 1902a: 258); *Epiperipatus biolleyi* (Peck 1975: 345).

Holotype: Not designated (see Remarks).

Type locality: Costa Rica, environs of San José (see Remarks).

Language of species description: French.

Remarks: Holotype not clearly designated in the original description. According to Bouvier (1907a: 519), a type has been deposited in the Museum National d’Histoire Naturelle de Paris, France. Specimens that match the original description occur in Las Nubes-Cascajal de Coronado, near San José, Costa Rica (e.g., Monge-Nájera et al. 1993; Mora et al. 1996; Mayer 2006). We regard *Epiperipatus biolleyi* and *Epiperipatus betheli* as separate species due to the great distance (808 km) between the type localities of the two species. Requires revision.

10. *Epiperipatus brasiliensis* (Bouvier, 1899a)

Synonyms: *Peripatus santarem* (senior synonym; Sedgwick 1888: 484; see Remarks); *Peripatus brasiliensis*, by original designation (Bouvier 1899a: 1031); *Epiperipatus brasiliensis brasiliensis* (Peck 1975: 345).

Holotype: Not designated (see Remarks).

Type locality: Brazil, Pará, Santarém.

Language of species description: French.

Remarks: The name *Peripatus brasiliensis* is a junior synonym of *Peripatus santarem* (Sedgwick 1888: 484). Sedgwick (1888: 484) referred to a specimen labelled “*Peripatus Santarem*, Wickham, purchased of W. H. J. Carter”, but it is uncertain whether “Santarem” represents a species name or the locality name on the label. Due to this uncertainty and due to the long usage of the name “*brasiliensis*”, we favour the latter name (ICZN Art. 11.6.1). For the sake of stability, we therefore consider *Peripatus santarem* as a *nomen oblitum* and *Peripatus brasiliensis* as a *nomen protectum*, following the ICZN (Art. 23). The holotype has not been clearly designated in the original description. According to Bouvier (1905: 270), the first specimens of this species collected by W.H.J. Carter and placed in the Natural History Museum of London are not type specimens as the author used other specimens from the same collection for species description. The species name is commonly misspelt as *braziliensis* (e.g., Arnett 1947; Eakin and Brandenburger 1966). We consider *Epiperipatus brasiliensis* as a separate and valid species due to the great distance between the type localities of the two putative subspecies (*brasiliensis* and *vagans*). Requires revision.

11. *Epiperipatus broadwayi* (Clark, 1913a)

Synonyms: *Peripatus (Epiperipatus) trinidadensis* var. *broadwayi* (Clark 1913a: 255); *Epiperipatus trinidadensis broadwayi* (Peck 1975: 346); *Epiperipatus broadwayi* (Read 1988b: 245).

Holotype: Not designated.

Type locality: Tobago Island. The precise locality might be the Tobago Forest Reserve (see Remarks).

Language of species description: Italian.

Remarks: The precise type locality is not provided in the original description (Clark 1913a: 255). The data provided by Read (1988b: 245) suggest that the species occurs in the area between Scarborough and the forested eastern end of the island, which currently belongs to the Tobago Forest Reserve. See Read (1988b) for further details.

12. *Epiperipatus cratensis* Brito, Pereira, Ferreira, Vasconscellos & Almeida, 2010

Synonyms: None.

Holotype: Deposited in the invertebrate collection of the Universidade Regional do Cariri, Crato, Brazil.

Type locality: Brazil, Ceará, Crato, Rio Batateiras, 07°16’S, 39°26’W.

Language of species description: English.

Remarks: Requires revision.

13. *Epiperipatus diadenoproctus* Oliveira, Lacorte, Fonseca, Wieloch & Mayer,2011

Synonyms: None.

Holotype: Deposited in the Instituto de Ciências Biológicas da Universidade Federal de Minas Gerais, Belo Horizonte, Brazil.

Type locality: BRAZIL, Minas Gerais, Simonésia, RPPN (=Particular Reserve of Natural Patrimony) Mata do Sossego, 20°04'21"S, 42°04'12"W, 1,150 m.

Language of species description: English.

Remark: The species diagnosis contains morphological and molecular characters (Oliveira et al. 2011).

14. *Epiperipatus edwardsii* (Blanchard, 1847)

Synonyms: *Peripatus edwardsii*, by original designation (Blanchard 1847: 140); *Peripatus (Epiperipatus) edwardsii* (Clark 1913b: 18); *Epiperipatus edwardsii* (Peck 1975: 345).

Holotype: Not designated (see Remarks).

Type locality: FRENCH GUIANA, Cayenne (see Remarks).

Language of species description: French.

Remarks: According to Bouvier (1907a: 519), a type has been deposited in the Museum National d’Histoire Naturelle de Paris, France. The species name is commonly misspelt as *edwardsi* (e.g., Bouvier 1905: 162; Peck 1975: 345). The species is known from the type locality, but subsequent records from an area extending from Brazil to Panama suggest that *Epiperipatus edwardsii* comprises a species complex (Read 1988b: 251, 253). Thus, a thorough revision of this putative species complex is required.

15. *Epiperipatus evansi* (Bouvier, 1904a)

Synonyms: *Peripatus evansi*, by original designation (Bouvier 1904a: 54); *Peripatus (Epiperipatus) evansi* (Clark 1913b: 18); *Epiperipatus evansi* (Peck 1975: 345).

Holotype: Not designated.

Type locality: British Guyana, Maccasseema on Pomeroon River.

Language of species description: French.

Remark: Requires revision.

16. *Epiperipatus hilkae* Morera-Brenes & Monge-Nájera, 1990

Synonyms: None.

Holotype: Deposited in the Museo de Zoologia de la Universidad de Costa Rica, San José, Costa Rica.

Type locality: COSTA RICA, Nicoya, Guanacaste, Parque Nacional Barra Honda, Bosque de las Cascadas, 10°11'N, 85°20'W, 200 m (see Remarks).

Language of species description: English.

Remarks: The species description was based on specimens from different localities (62 km apart from each other). Thus, it might comprise a species complex, which requires revision.

17. *Epiperipatus imthurni* (Sclater, 1888)

Synonyms: *Peripatus demeraranus* (junior synonym; Sedgwick 1888: 476; see Remarks); *Peripatus imthurni*, by original designation (Sclater 1888: 344); *Peripatus (Epiperipatus) imthurmi* (Clark 1913b: 18); *Epiperipatus imthurmi* (Peck 1975: 345).

Holotype: Not designated.

Type locality: British Guyana, Maccaseema on Pomeroon river (see Remarks).

Language of species description: English.

Remarks: The species name is commonly misspelt as *imthurmi* (e.g., Clark 1913b: 18; Peck 1975: 345). The name *Peripatus demeraranus* is considered as a junior synonym because the corresponding species description was published in March 1888, i.e., one month later than the description of *Peripatus imthurni*. The name *Peripatus demeraranus* is regarded here as a *nomen nudum*. The original description has imprecise locality data. In this context, the name Demerara may be assigned either to the river Demerara or to the Demerara region (previous name of British Guyana). However, the specimens analysed by Sedgwick (1888: 474) seem to be the same studied by Sclater (1888), which were collected at “Maccaseema on Pomeroon River”. This species name has been assigned also to specimens from other localities, including Trinidad (Read 1988b: 241). Thus, *Epiperipatus imthurni* might comprise a species complex, which requires revision.

18. *Epiperipatus isthmicola* (Bouvier, 1902b)

Synonyms: *Peripatus nicaraguensis* var. *isthmicola*, by original designation (Bouvier 1902b: 240); *Peripatus (Epiperipatus) isthmicola* (Clark 1913b: 18); *Epiperipatus isthmicola* (Peck 1975: 345).

Holotype: Not designated (see Remarks).

Type locality: COSTA RICA, Cartago, environs of San José (see Remarks).

Language of species description: French.

Remarks: The holotype has not been clearly designated in the original description. According to Bouvier (1907a: 519), a type has been deposited in the Museum National d’Histoire Naturelle de Paris, France. The description contains imprecise type locality data. A redescription of this species (Bouvier 1905) was based on specimens from different localities, suggesting that it comprises a species complex, which requires revision.

19. *Epiperipatus lewisi* Arnett, 1961

Synonyms: None.

Holotype: Deposited in the Smithsonian National Museum of Natural History, Washington D.C., USA.

Type locality: JAMAICA, Portland, John Crow Mountains, ca. 16 km (10 miles) southwest of Priestman’s river.

Language of species description: English.

Remark: Requires revision.

20. *Epiperipatus machadoi* (Oliveira & Wieloch, 2005)

Synonyms: *Macroperipatus machadoi*, by original designation (Oliveira and Wieloch 2005: 61); *Epiperipatus machadoi* (Oliveira et al. 2010: 25).

Holotype: Deposited in the Instituto de Ciências Biológicas da Universidade Federal de Minas Gerais, Belo Horizonte, Brazil.

Type locality: BRAZIL, Minas Gerais, Caratinga, RPPN Feliciano Miguel Abdala, 19°43'52"S, 41°49'02"W, 410 m.

Language of species description: Portuguese.

21. *Epiperipatus paurognostus* Oliveira, Lacorte, Fonseca, Wieloch & Mayer,2011

Synonyms: *Epiperipatus schedocrypticus* (*nomen nudum*; Lacorte et al. 2010: 342; see Remarks).

Holotype: Deposited in the Instituto de Ciências Biológicas da Universidade Federal de Minas Gerais, Belo Horizonte, Brazil.

Type locality: BRAZIL, Minas Gerais, Piedade de Caratinga, Mata do Eremitério, 19°45'33"S, 42°05'22"W, 897 m.

Language of species description: English.

Remarks: The name *Epiperipatus schedocrypticus* (Lacorte et al. 2010) is invalid according to the ICZN since it was suggested without providing a formal description (Art. 13) and without depositing type specimens (Art. 16). Moreover, according to the ICZN (Art. 9.9), the proposition of this name in a proceedings abstract cannot be regarded as a valid publication for purposes of zoological nomenclature. *Epiperipatus paurognostus* is very similar morphologically to *Epiperipatus adenocryptus*. The species diagnosis contains morphological and molecular characters (Oliveira et al. 2011).

22. *Epiperipatus simoni* (Bouvier, 1899b)

Synonyms: *Peripatus simoni*, by original designation (Bouvier 1899b: 271); *Peripatus (Epiperipatus) simoni* (Clark 1913b: 18); *Epiperipatus simoni* (Peck 1975: 346).

Holotype: Not designated (see Remarks).

Type locality: VENEZUELA, Caracas (see Remarks).

Language of species description: French.

Remarks: The holotype has not been clearly designated in the original description. According to Bouvier (1907a: 519), a type has been deposited in the Museum National d’Histoire Naturelle de Paris, France. A more precise locality for the species might be in the San Esteban National Park, near Caracas, according to Bouvier (1905: 221). The species name has been misspelt as *F. semoni* [sic] by Vasconcellos et al. (2004: 140). Requires revision.

23. *Epiperipatus torrealbai* Scorza, 1953

Synonyms: None.

Holotype: Deposited in the Museo de Zoologia de la Universidad Central de Venezuela, Caracas, Venezuela.

Type locality: VENEZUELA, Los Chorros, near Caracas.

Language of species description: Spanish.

Remarks: Requires revision.

24. *Epiperipatus trinidadensis* (Sedgwick, 1888)

Synonyms: *Peripatus trinidadensis*, by original designation (Sedgwick 1888: 477); *Peripatus trinitatis* (Bouvier 1905: 289); *Peripatus (Epiperipatus) trinidadensis* (Clark 1913b: 18); *Epiperipatus trinidadensis* (Peck 1975: 346).

Holotype: Not designated.

Type locality: TRINIDAD (see Remarks).

Language of species description: English.

Remarks: An incorrect authority (Stuhlmann) is commonly attributed to this species (e.g., Bouvier 1905: 290; Peck 1975: 346), although the corresponding publication does not contain the species name (Stuhlmann 1886). Precise data on the type locality have not been provided in the original description. The species has been re-described by Read (1988b: 247), who recorded the species from different localities, all in the Northern Range of Trinidad, including a large number of specimens from the Simla Research Station, 6.4 km (4 miles) north of Arima (Read 1988b: 248). Thus, the species might comprise a species complex, which requires revision.

25. *Epiperipatus vagans* (Brues, 1925)

Synonyms: *Peripatus (Epiperipatus) brasiliensis* var. *vagans*, by original designation (Brues 1925: 162); *Epiperipatus brasiliensis vagans* (Peck 1975: 345).

Holotype: Deposited in the Smithsonian National Museum of Natural History, Washington D.C., USA.

Type locality: Panama, Canal Zone, Barro Colorado.

Language of species description: English.

Remarks: We regard *Epiperipatus vagans* and *Epiperipatus brasiliensis* as separate species rather than subspecies, due to the great distance between their type localities (3,066 km). Revision is required as it might reveal morphological and molecular differences between *Epiperipatus vagans* and *Epiperipatus brasiliensis*.

26. *Epiperipatus vespuccii* Brues, 1914

Synonyms: *Peripatus (Epiperipatus) vespuccii*, by original designation (Brues 1914: 375); *Epiperipatus vespuccii* (Peck 1975: 346).

Holotype: Deposited in the Museum of Comparative Zoology at Harvard University, Cambridge, USA.

Type locality: COLOMBIA, Magdalena, Sierra Nevada de Santa Marta, Cincinnati Coffee Plantation, 701 m (2,300 ft).

Language of species description: English.

Remarks: Requires revision.

***Nomina dubia***

*Epiperipatus nicaraguensis* (Bouvier, 1900a)

Synonyms: *Peripatus nicaraguensis*, by original designation (Bouvier 1900a: 395); *Peripatus (Epiperipatus) nicaraguensis* (Clark 1913b: 18); *Epiperipatus nicaraguensis* (Peck 1975: 346).

Holotype: Not designated (see Remarks).

Type locality: NICARAGUA, Matagalpa (see Remarks).

Language of species description: French.

Remarks: The holotype has not been clearly designated in the original description. Röhlig et al. (2010: 227) refer to a holotype placed in the Museum für Naturkunde Berlin, which should be regarded as a syntype instead. The description contains imprecise type locality data (Matagalpa in Nicaragua occupies an area of 8,523 km^2^). Bouvier (1905: 327) stated that a specimen collected by Belt (1874: 140) might belong to this species. However, this record is doubtful since the author might have misinterpreted the information provided by Belt (1874: 140), who referred to San Benito Mine located in Santo Domingo rather than to San Benito located in San Antonio Valley (see Bouvier 1905: 328). Revision of this species will be difficult since no precise locality data are available in the literature.

*Epiperipatus tucupi* (Froehlich, 1968)

Synonyms: *Peripatus (Epiperipatus) tucupi*, by original designation (Froehlich 1968: 168); *Epiperipatus tucupi* (Peck 1975: 346).

Holotype: Deposited in the Museu de Zoologia da Universidade de São Paulo, São Paulo, Brazil.

Type locality: BRAZIL, Pará (see Remarks).

Language of species description: English.

Remarks: The description contains imprecise type locality data (the Pará State of Brazil occupies an area of 1,247,689.515 km^2^ within Amazonia). Since no further work with more precise locality data is available, a revision of this species based on topotypes will be difficult.

##### *Heteroperipatus* Zilch, 1954a

Type species: *Heteroperipatus engelhardi* Zilch, 1954a, by original designation (Zilch 1954a: 148).

27. *Heteroperipatus clarki* (Dunn, 1943)

Synonyms: *Peripatus clarki*, by original designation (Dunn 1943: 2); *Heteroperipatus clarki* (Zilch 1954a: 148, in a footnote).

Holotype: Deposited in the collection of the Academy of Natural Sciences of Philadelphia, Philadelphia, USA.

Type locality: PANAMA, Azuero Peninsula, province of Veragua, north base of the ridge supporting Piedra del Tigre, near western border of Veragua, “two days south of Las Minas” (according to Dunn 1943), 792 m (2,600 ft).

Language of species description: English.

Remark: Note that the name *clarki* has also been used by Arnett (1961) for *Macroperipatus insularis clarki* (see Synonyms for *Macroperipatus clarki* below). The species might have been included in *Heteroperipatus* based on an ambiguous character and, therefore, requires revision.

28. *Heteroperipatus engelhardi* Zilch, 1954a

Synonyms: None.

Holotype: Likely to have been deposited in the Senckenberg Research Institute and Natural History Museum, Frankfurt, Germany, as the author was employed at this institution at that time and used the acronym SMF in his description (Zilch 1954a: 150).

Type locality: EL SALVADOR, San Vicent, Finca El Carmen, San Vicent Vulcano (Las Chiches), 1,300 m.

Language of species description: German.

Remark: Requires revision.

##### *Macroperipatus* (Clark, 1913b)

Type species: *Macroperipatus torquatus* (von Kennel, 1883), by original designation (Clark 1913b: 17).

Remarks: As for *Epiperipatus*. Most species might have been assigned to *Macroperipatus* based on a fixation artefact (Oliveira et al. 2010: 31) and *Macroperipatus torquatus* might be the only species belonging to the (monotypic) genus. The entire genus requires revision (Oliveira et al. 2010).

29. *Macroperipatus clarki* Arnett, 1961

Synonyms: *Macroperipatus insularis clarki*,by original designation (Arnett 1961: 215).

Holotype: Deposited in the Science Museum of the Institute of Jamaica, Kingston, Jamaica.

Type locality: JAMAICA, Portland, John Crow Mountains, ca. 8 km (5 miles) southwest of the Priestman’s River, 457 m (ca. 1,500 ft).

Language of species description: English.

Remarks: Note that Dunn (1943) used the name *clarki* for *Peripatus clarki* (see Synonyms of *Heteroperipatus clarki* above). We regard *Macroperipatus clarki* and *Macroperipatus insularis* as separate species rather than subspecies due to the great distance between their type localities (430 km) on different islands. Revision is required as it might reveal morphological and molecular differences between *Macroperipatus clarki* and *Macroperipatus insularis*.

30. *Macroperipatus guianensis* (Evans, 1903)

Synonyms: *Peripatus guianensis*, by original designation (Evans 1903: 145); *Peripatus ohausi* var. *guianensis* (Bouvier 1904a: 53); *Peripatus (Macroperipatus) guianensis* (Clark 1913b: 17); *Macroperipatus guianensis* (Peck 1975: 346).

Holotype: Not designated (see Remarks).

Type locality: BRITISH GUIANA, Demerara-Haimaca, eastern bank of the river Demerara.

Language of species description: English.

Remarks: The holotype has not been designated explicitly, but the author used the terms “male type specimen” and “female type specimen” in his figure legends (Evans 1903: 159–160). Requires revision.

31. *Macroperipatus insularis* Clark, 1937

Synonyms: *Macroperipatus insularis insularis* (Peck 1975: 347).

Holotype: Deposited in the Smithsonian National Museum of Natural Science, Washington D.C., USA.

Type locality: HAITI, between Jacmel and Tronin (see Remarks).

Language of species description: English.

Remarks: The current name and geographical position of Tronin could not be found. We regard *Macroperipatus insularis* and *Macroperipatus clarki* as separate species rather than subspecies due to the great distance between their type localities (430 km) situated on different islands. Revision is required as it might reveal morphological and molecular differences between *Macroperipatus clarki* and *Macroperipatus insularis*.

32. *Macroperipatus ohausi* (Bouvier, 1900b)

Synonyms: *Peripatus ohausi*, by original designation (Bouvier 1900b: 67); *Peripatus (Macroperipatus) ohausi* (Clark 1913b: 17); *Macroperipatus ohausi* (Peck 1975: 347).

Holotype: Not designated.

Type locality: BRAZIL, Rio de Janeiro, Petrópolis.

Language of species description: French.

Remarks: Although the holotype has not been clearly designated in the original description, Weidner (1959: 93) refers to an holotype found in the Zoologisches Staatsinstitut und Zoologisches Museum Hamburg, Hamburg, Germany. Requires revision.

33. *Macroperipatus perrieri* (Bouvier, 1899c)

Synonyms: *Peripatus perrieri*, by original designation (Bouvier 1899c: 1345); *Peripatus (Macroperipatus) perrieri* (Clark 1913b: 17); *Macroperipatus perrieri* (Peck 1975: 347).

Holotype: Not designated (see Remarks).

Type locality: MEXICO, Vera Cruz.

Language of species description: French.

Remarks: The holotype has not been designated explicitly in the original description. According to Bouvier (1907a: 518), a type has been deposited in the Museum National d’Histoire Naturelle de Paris, France. Requires revision.

34. *Macroperipatus torquatus* (von Kennel, 1883)

Synonyms: *Peripatus torquatus*, by original designation (von Kennel 1883: 532); *Peripatus (Macroperipatus) torquatus* (Clark 1913b: 17); *Macroperipatus torquatus* (Peck 1975: 347).

Holotype: Not designated.

Type locality: TRINIDAD. The precise locality is unknown, but Read (1988b) states that all confirmed records of this species are from the Northern Range (see Remarks).

Language of species description: German.

Remarks: The specimens of *Macroperipatus torquatus* held in the Natural History Museum of London, United Kingdom, were collected near Mount Aripo, east of Arima, 122 m (400 ft), which might be a more precise locality for the species on the island of Trinidad. Requires revision.

35. *Macroperipatus valerioi* Morera-Brenes & Léon, 1986

Synonyms: None.

Holotype: Deposited in the Museo de Insecto de la Universidad de Costa Rica, Costa Rica.

Type locality: COSTA RICA, Rio Damitas, 16 km north of Puerto Quepos, 9°34'N, 84°10'W, 600 m.

Language of species description: English.

Remarks: Requires revision.

***Nomen dubium***

*Macroperipatus geayi* (Bouvier, 1899c)

Synonyms: *Peripatus geayi*, by original designation (Bouvier 1899c: 1345); *Peripatus (Macroperipatus) geayi* (Clark 1913b: 17); *Macroperipatus geayi* (Peck 1975: 246).

Holotype: Not designated (see Remarks).

Type locality: BRAZIL, Carsevenne (=Calçoene) (high Carsevenne according to the type specimen label; see Remarks).

Language of species description: French.

Remarks: The holotype has not been designated explicitly in the original description. According to Bouvier (1905: 201; 1907a: 518), a type has been deposited in the Museum National d’Histoire Naturelle de Paris, France. The species name was misspelt as *geagy* by Jerez-Jaimes and Bernal-Pérez (2009: 567) and as *geagi* by Morera-Brenes and Léon (1986: 278). The description contains imprecise type locality data (the region of Calçoene in the Amapá State occupies 14,269 km^2^). The information found in the type specimen label might refer to the river with the same name, which crosses the region. Since no further work with more precise locality data is available, a revision of this species based on topotypes will be difficult.

##### *Mesoperipatus* Evans, 1901a

Type species: *Mesoperipatus tholloni* (Bouvier, 1898a), by subsequent monotypy.

36. *Mesoperipatus tholloni* (Bouvier, 1898a)

Synonyms: *Peripatus tholloni*, by original designation (Bouvier 1898a: 1359); *Mesoperipatus tholloni* (Evans 1901a: 478).

Holotype: Not designated (see Remarks).

Type locality: GABON. It might be in Ngolé, on the river Ogowe (see Remarks).

Language of species description: French. English translation available (Bouvier 1898b).

Remarks: The holotype has not been designated explicitly in the original description. According to Bouvier (1905: 349; 1907a: 519), a type has been deposited in the Museum National d’Histoire Naturelle de Paris, France. The original description and the putative type contain imprecise type locality data (Gabon occupies an area of 267,667 km^2^). However, Bouvier (1905: 348) refers to additional specimens found by M. Haug in Ngolé (spelt Ngômô) along the river Ogowe (spelt Ogôoué) and discusses that this might be the more precise locality for the species since the label of the type says only “Gabon” (a common practice at that time according to Bouvier 1905). Bouvier (1907a: 519) refers also to an additional specimen of this species collected in Ndjolé, along the same river. Therefore, it cannot be ruled out that *Mesoperipatus tholloni* is a species complex, which requires revision.

##### *Oroperipatus* (Cockerell, 1908)

Type species: *Oroperipatus lankesteri* (Bouvier, 1899a), by original designation (Cockerell 1908: 620).

Remarks: The name was introduced initially as a subgenus of *Peripatus* (see Cockerell 1908: 620). It was raised to genus status by Clark (1913b), who stated: “It seems advisable, therefore, to recognize these smaller units as in reality of generic rank” (Clark 1913b: 16).

37. *Oroperipatus balzani* (Camerano, 1897)

Synonyms: *Peripatus balzani*, by original designation (Camerano 1897: 14); *Oroperipatus balzani* (Clark 1913b: 16).

Holotype: Not designated.

Type locality: BOLIVIA, Yungas, Chulumani, near Coroico, 1,600 m.

Language of species description: Italian.

Remark: Requires revision.

38. *Oroperipatus belli* (Bouvier, 1904b)

Synonyms: *Peripatus belli*, by original designation (Bouvier 1904b: 56); *Oroperipatus belli* (Clark 1913b: 16).

Holotype: Not designated.

Type locality: ECUADOR, Durán, Guayras river (misspelling of Guayas river), opposite to Guayaquil (according to the label of the putative type specimens).

Language of species description: French.

Remark: Requires revision.

39. *Oroperipatus bimbergi* (Fuhrmann, 1913)

Synonyms: *Peripatus bimbergi*, by original designation (Fuhrmann 1913: 242); *Oroperipatus bimbergi* (Clark 1915: 14).

Holotype: Not designated.

Type locality: COLOMBIA, Amagatal in the central mountain range (900–1,800 m) and Guaduas (800 m) towards Bogota, in the eastern mountain range (see Remarks).

Language of species description: German.

Remark: The current location of Amagatal could not be found. Requires revision.

40. *Oroperipatus bluntschlii* Fuhrmann, 1915

Synonyms: none (see Remarks).

Holotype: Not designated.

Type locality: PERU, Loreto, Shapajilla, Samiria River, 120 m.

Language of species description: German.

Remarks: In addition to *Oroperipatus bluntschlii*, the author used the name *Peripatus bluntschlii* in the original description (Fuhrmann 1915: 35). Requires revision.

41. *Oroperipatus cameranoi* (Bouvier, 1899a)

Synonyms: *Peripatus cameranoi*, by original designation (Bouvier 1899a: 1030); *Oroperipatus cameranoi* (Clark 1913b: 16).

Holotype: Not designated.

Type locality: ECUADOR, Azuay, Sigsig, southeast of Cuenca, 2,550 m.

Language of species description: French.

Remark: Requires revision.

42. *Oroperipatus corradoi* (Camerano, 1898)

Synonyms: *Peripatus corradi*, by original designation (Camerano 1898: 310; see Remarks); *Peripatus corradoi* (Bouvier 1905: 20; see Remarks); *Oroperipatus corradoi* (Clark 1913b: 16).

Holotype: Not designated.

Type locality: ECUADOR, Pichincha, environs of Quito.

Language of species description: Italian.

Remarks: The correct spelling of the species name should be *corradoi*, following the etymology of the original description, which says: “*lieto di dedicarla all’AVv. Corrado Festa padre del nostro coraggioso e generoso esploratore e naturalista il Dott. Enrico Festa*” (Camerano 1898: 310)[loose English translation: “I am happy to dedicate it (the species name) to the lawyer Corrado Festa, the father of our courageous and generous explorer and naturalist Dr. Enrico Festa”]. Following the ICZN (Art. 32.5.1), we suggest the above modification and consider, from now onwards, *corradi* as a misspelling of the species name. The description contains imprecise locality data and the redescription of the species (Bouvier 1905: 120) was based on specimens from different localities, suggesting that it might be a species complex, which, thus, requires revision.

43. *Oroperipatus ecuadorensis* (Bouvier, 1902c)

Synonyms: *Peripatus ecuadorensis*, by original designation (Bouvier 1902c: 53); *Oroperipatus equadoriensis* (Clark 1913b: 16; see Remarks).

Holotype: Deposited in the Museum National d’Histoire Naturelle de Paris, France.

Type locality: ECUADOR, Bulim (current Púlun), northwestern Ecuador, Pacific side of the Andes, 18 m (60 ft).

Language of species description: French.

Remarks: The species name is commonly misspelt as *equadoriensis* (e.g., Clark 1913b: 16) or *ecuadoriensis* (e.g., Clark 1915: 25; Peck 1975: 347). Requires revision.

44. *Oroperipatus eisenii* (Wheeler, 1898)

Synonyms: *Peripatus eisenii*, by original designation (Wheeler 1898: 1); *Oroperipatus eiseni* (Clark 1913b: 16; see Remarks).

Holotype: Not designated.

Type locality: MEXICO, Nayarit, outskirts of Tepic, 1,219 m (4,000 ft).

Language of species description: English.

Remarks: The species name is commonly misspelt as *eiseni* (e.g., Clark 1913b: 16; Vasconcellos et al. 2004: 140). A note on putative specimens of this species was published recently by Cupul-Maganã and Navarrete-Heredia (2008), but it is based on specimens from Puerto Vallarta (Jalisco, Mexico), which is situated 100 km away from the type locality. Requires revision.

45. *Oroperipatus intermedius* (Bouvier, 1901a)

Synonyms: *Peripatus intermedius*, by original designation (Bouvier 1901a: 168); *Oroperipatus intermedius* (Clark 1913b: 16).

Holotype: Deposited in the Natural History Museum of Lübeck, Lübeck, Germany.

Type locality: BOLIVIA, La Paz, Sorata.

Language of species description: French.

Remark: Requires revision.

46. *Oroperipatus koepckei* Zilch, 1954b

Synonyms: None.

Holotype: Likely to have been deposited in the Senckenberg Research Institute and Natural History Museum, Frankfurt, Germany, as the author was employed at this institution at that time and used the acronym “SMF” in his description (Zilch 1954b: 153).

Type locality: PERU. The precise locality might be in the Piura province, “35 km from Olmos toward Jaén, ca. 6°10'S, 79°30'W, ca. 1,400 m” according to Zilch (1954b: 153).

Language of species description: German.

Remark: Requires revision.

47. *Oroperipatus lankesteri* (Bouvier, 1899a)

Synonyms: *Peripatus lankesteri*, by original designation (Bouvier 1899a: 1030); *Oroperipatus lankesteri* (Clark 1913b: 16).

Holotype: Not designated.

Type locality: ECUADOR, Imbabura, river Parambas, 16 km (10 miles) north of Quito (according to the label of the type specimens).

Language of species description: French.

Remark: Requires revision.

48. *Oroperipatus multipodes* (Fuhrmann, 1913)

Synonyms: *Peripatus multipodes*, by original designation (Fuhrmann 1913: 244); *Oroperipatus multipodes* (Clark 1915: 25).

Holotype: Not designated.

Type locality: COLOMBIA, Antioqui, Concordia, river Amagá (see Remarks).

Language of species description: German.

Remarks: The name of the river might have been misspelt in the original description as “Rio Amago” (Fuhrmann 1913: 244). Requires revision.

49. *Oroperipatus omeyrus* du Bois-Reymond Marcus, 1952

Synonyms: None.

Holotype: Not designated.

Type locality: PERU, Cusco, Sahuayaco in the Urubamba Valley (between Abancay and Maras), 800 m and San José de Lourdes (Cajamarca), 1000 m (see Remarks).

Language of species description: English.

Remarks: Note that du Bois-Reymond Marcus is the full name of the author, whereas commonly cited Marcus, 1952 is incomplete. The species description was based on specimens from different localities (1084 km apart from each other). Thus, it might comprise a species complex, which requires revision.

50. *Oroperipatus peruvianus* (Brues, 1917)

Synonyms: *Peripatus (Oroperipatus) peruvianus*, by original designation (Brues 1917: 383); *Oroperipatus peruvianus* (du Bois-Reymond Marcus, 1952: 191).

Holotype: Deposited in the Museum of Comparative Zoology at Harvard University, Cambridge, USA.

Type locality: PERU, Cajamarca, Tabaconas, near Huancabamba, 1,829 m (6,000 ft).

Language of species description: English.

Remark: Requires revision.

51. *Oroperipatus soratanus* (Bouvier, 1901a)

Synonyms: *Peripatus soratanus*, by original designation (Bouvier 1901a: 168); *Oroperipatus soratanus* (Clark 1913b: 16).

Holotype: Not designated.

Type locality: BOLIVIA, La Paz, Sorata.

Language of species description: French.

Remark: Requires revision.

52. *Oroperipatus tuberculatus* (Bouvier, 1898c)

Synonyms: *Peripatus tuberculatus*, by original designation (Bouvier 1898c: 1525); *Oroperipatus tuberculatus* (Clark 1913b: 16).

Holotype: Not designated (see Remarks).

Type locality: COLOMBIA, Cauca, Popayán.

Language of species description: French. English translation available (Bouvier 1898d).

Remarks: The holotype has not been designated explicitly in the original description. According to Bouvier (1907a: 518), a type has been deposited in the Museum National d’Histoire Naturelle de Paris, France. Requires revision.

53. *Oroperipatus weyrauchi* du Bois-Reymond Marcus, 1952

Synonyms: None.

Holotype: Not designated.

Type locality: PERU, Yurac, River Aguaytía, left affluent of the Ucayali.

Language of species description: English.

Remarks: As for *Oroperipatus omeyrus*, except that this species was recorded from a single locality. Requires revision.

**Nomina dubia**

*Oroperipatus goudoti* (Bouvier, 1899c)

Synonyms: *Peripatus goudoti*, by original designation (Bouvier 1899c: 1345); *Oroperipatus goudoti* (Clark 1913b: 16).

Holotype: Not designated (see Remarks).

Type locality: MEXICO.

Language of species description: French.

Remarks: The holotype has not been designated explicitly in the original description. According to Bouvier (1907a: 518), a type has been deposited in the Museum National d’Histoire Naturelle de Paris, France. The description contains imprecise type locality data (Mexico occupies an area of 1,972,550 km^2^). Since no further work with more precise locality data is available, a revision of this species based on topotypes will be difficult.

*Oroperipatus quitensis* (Schmarda, 1871)

Synonyms: *Peripatus quitensis*, by original designation (Schmarda 1871: 76; see Remarks); *Oroperipatus quitensis* (Clark 1913b: 16).

Holotype: Not designated (see Remarks).

Type locality: ECUADOR, “equatorial highlands of South America” (see Remarks).

Language of species description: German.

Remarks: Information on species description was obtained from the second edition of Schmarda’s text book (Schmarda 1878: 74–77) and from Bell (1887). Although the species name suggests that the species locality is the environs of Quito, neither data on precise type locality nor information on type specimens is found in the literature. Since no further work with more precise locality data is available, a revision of this species based on topotypes will be difficult.

*Oroperipatus peruanus* (Grube, 1876)

Synonyms: *Peripatus peruanus*, by original designation (Grube 1876: 72); *Oroperipatus peruanus* (Peck 175: 348).

Holotype: Not designated.

Type locality: PERU.

Language of species description: German.

Remarks: We regard the species as a *nomen dubium* due to the lack of precise information on its morphology, type designation and type locality. The species has been regarded as doubtful by several authors (e.g., Bouvier 1905: 74; 1907b: 300; du Bois-Reymond Marcus 1952: 192; Zilch 1954b: 151).

##### *Peripatus* Guilding, 1826

Type species: *Peripatus juliformis* Guilding, 1826, by subsequent monotypy.

Remark: This oldest genus is referred to by some authors as *Peripatus sensu stricto* (e.g., Clark 1913b; Froehlich 1962).

54. *Peripatus basilensis* Brues, 1935

Synonyms: *Peripatus dominicae* var. *basilensis*, by original designation (Brues 1935: 62); *Peripatus dominicae basilensis* (Peck 1975: 348).

Holotype: Not designated.

Type locality: HAITI, Morne Basile (Mount Basil), northwestern part of the island (Brues 1939: 36).

Language of species description: English.

Remarks: We regard *Peripatus dominicae*, *Peripatus basilensis, Peripatus darlingtoni*, *Peripatus haitiensis and Peripatus lachauxensis* as separate species rather than subspecies due to great distances between their type localities (ranging from 115 km to 1,380 km). Although the type localities of *Peripatus lachauxensis* and *Peripatus darlingtoni* are relatively close to each other (7 km), their status as separate species is supported by morphological differences and by their occurrences at different altitudes (*Peripatus lachauxensis*: 305 m; *Peripatus darlingtoni*: 914 m) and in different environments (Brues 1935: 61–62). *Peripatus basilensis* has also been recorded from additional localities (Brues 1939: 36), indicating that it might be a species complex. Revision of these five species is required, which might reveal additional morphological and molecular differences between them.

55. *Peripatus bouvieri* Fuhrmann, 1913

Synonyms: None.

Holotype: Not designated.

Type locality: COLOMBIA, Boca del Monte, at the border between Casanare and Arauca.

Language of species description: German.

Remarks: Note that the name *bouvieri* has been used by Cockerell (1901: 326) for *Peripatus jamaicensis* mut. *bouvieri* (see *Plicatoperipatus jamaicensis* below). Requires revision.

56. *Peripatus broelemanni* Bouvier, 1899c

Synonyms: None.

Holotype: Not designated (see Remarks).

Type locality: VENEZUELA, Mérida, Tovar.

Language of species description: French.

Remarks: The holotype has not been designated explicitly in the original description. According to Bouvier (1907a: 519), a type has been deposited in the Museum National d’Histoire Naturelle de Paris, France. The species name has been spelt as *Peripatus brölemanni* in the original description (Bouvier 1899c: 1345); *brolemanni* is a common misspelling (e.g., Peck 1975: 348). Requires revision.

57. *Peripatus danicus* Bouvier, 1900c

Synonyms: *Peripatus juliformis danicus*, by original designation (Bouvier 1900c: 751; see Remarks); *Peripatus danicus* (Clark, 1913b: 17).

Holotype: Not designated.

Type locality: VIRGIN ISLANDS, Saint Thomas Island.

Language of species description: French.

Remarks. Peck (1975: 348) used the old species name suggested by Bouvier rather than that suggested by Clark (1913b: 17). The description contains imprecise type locality data. However, the area is relatively small (Saint Thomas Island occupies an area of 81 km^2^) and clearly separate from other islands and from the mainland by seawater. Hence, we regard *Peripatus danicus* as a valid species, although we cannot rule out that additional species might be found on the same island. A revision of this species, including specimens from different localities on the island, is required.

58. *Peripatus darlingtoni* Brues, 1935

Synonyms: *Peripatus dominicae* var. *darlingtoni*, by original designation (Brues 1935: 62); *Peripatus dominicae darlingtoni* (Peck 1975: 348).

Holotype: Not designated.

Type locality: HAITI, Massif (Plateau) de la Hotte, southwestern peninsula of Haiti, between Camp Perrion and Mafin, 914 m (3,000 ft).

Language of species description: English.

Remarks: As for *Peripatus basilensis*, except that this species was recorded from a single locality. Requires revision.

59. *Peripatus dominicae* Pollard, 1893

Synonyms: *Peripatus dominicae*, by original designation (Pollard 1893: 290); *Peripatus dominicae dominicae* (Peck 1975: 348).

Holotype: Not designated.

Type locality: DOMINICA ISLAND, Laudat (see Remarks).

Language of species description: English.

Remarks: As for *Peripatus basilensis*, except that this species was recorded from a single locality. Locality data obtained from specimens deposited in the Natural History Museum of London, United Kingdom. Requires revision.

60. *Peripatus evelinae* Marcus, 1937

Synonyms: *Peripatus (Epiperipatus) evelinae*, by original designation (Marcus 1937: 905); *Peripatus evelinae* (Peck 1975: 348).

Holotype: Not designated. The lectotype has been deposited in the Museu de Zoologia da Universidade de São Paulo, São Paulo, Brazil (Froehlich 1968: 160).

Type locality: BRAZIL, Goyas (=Goiás), in the area between Cana Brava (current Minaçu) and Nova Roma. The precise locality might be the environs of Nova Roma (Froehlich 1968: 160; see Remarks).

Language of species description: Portuguese.

Remarks: Although the original description contains imprecise data on the type locality (the two localities mentioned lie 162 km apart), redescription of this species provides more precise locality data (Froehlich 1968). Requires revision.

61. *Peripatus haitiensis* Brues, 1913

Synonyms: None.

Holotype: Deposited in the Museum of Comparative Zoology at Harvard University, Cambridge, USA.

Type locality: HAITI, Massif (Plateau) de la Selle, Furcy. The precise locality might be the La Visite National Park, 1,524–2,134 m (5,000–7,000 ft) (Brues 1935: 61).

Language of species description: English.

Remarks: As for *Peripatus basilensis*, except that this species was recorded from a single locality. Requires revision.

62. *Peripatus heloisae* Carvalho, 1941

Synonyms: None.

Holotype: Deposited in the Museu Nacional do Rio de Janeiro, Rio de Janeiro, Brazil.

Type locality: BRAZIL, Mato Grosso, left border of Tapirapé river, next to its confluence with the river Araguaia (See Remarks).

Language of species description: Portuguese.

Remarks: Although the species name was introduced in 1941, a comprehensive species description followed one year later (Carvalho 1942). The river Araguaia was misspelt as Araraguaia by Carvalho (1941: 448). According to Sampaio-Costa et al. (2009), the precise type locality might be Santa Terezinha, the current name for Barra do Tapirapé used by Carvalho (1942: 66). Requires revision.

63. *Peripatus juanensis* Bouvier, 1900a

Synonyms: *Peripatus dominicae juanensis*, by original designation (Bouvier 1900a: 394); *Peripatus juanensis* (Clark 1913b: 17).

Holotype: Not designated (see Remarks).

Type locality: PUERTO RICO, Utuado, Utuado.

Language of species description: French.

Remark: The holotype was not clearly designated in the original description. Röhlig et al. (2010: 227) refer to syntypes placed in the Museum für Naturkunde Berlin, Germany. Requires revision.

64. *Peripatus juliformis* Guilding, 1826

Synonyms: None.

Holotype: Not designated.

Type locality: SAINT VINCENT ISLAND. The precise locality might be the Grand Bonhomme Mountain (Guilding 1826: 444). The current name of the type locality might be Mt. Bonum (Read 1988b).

Language of species description: Latin.

Remark: A wrong year of description, 1825, has been commonly assigned to the species name (e.g. Bouvier 1905; Clark 1913b). Requires revision.

65. *Peripatus lachauxensis* Brues, 1935

Synonyms: *Peripatus dominicae* var. *lachauxensis*, by original designation (Brues 1935: 61); *Peripatus dominicae lachauxensis* (Peck 1975: 348).

Holotype: Not designated.

Type locality: HAITI, south eastern foothills of the Massif (Plateau) de la Hotte, southeastern peninsula of Haiti, 305 m (1,000 ft).

Language of species description: English.

Remarks: As for *Peripatus basilensis*, except that this species was recorded from a single locality. Requires revision.

66. *Peripatus manni* Brues, 1913

Synonyms: None.

Holotype: Deposited in the Museum of Comparative Zoology at Harvard University, Cambridge, USA.

Type locality: HAITI, Massif (Plateau) de la Selle. The precise locality might be La Visite National Park, 1,524–2,134 m (5,000–7,000 ft) (Brues 1935: 61)

Language of species description: English.

Remark: Requires revision.

67. *Peripatus ruber* Fuhrmann, 1913

Synonyms: None.

Holotype: Not designated.

Type locality: COSTA RICA, San José, Rancho Redondo.

Language of species description: German.

Remark: Requires revision.

68. *Peripatus sedgwicki* Bouvier, 1899c

Synonyms: None.

Holotype: Not designated.

Type locality: VENEZUELA, Federal district, environs of Caracas. The precise locality might be La Guaira (Bouvier 1905: 213).

Language of species description: French.

Remarks: Note that the abbreviation *Peripatus sedgwicki* may be confused with the same abbreviation of *Peripatopsis sedgwicki*, the junior synonym of *Peripatopsis dewaali* (Weber, 1898). The species redescription was based on specimens from different localities (Bouvier 1905: 220), indicating that *Peripatus sedgwicki* might be a species complex, which requires revision.

69. *Peripatus solorzanoi* Morera-Brenes & Monge-Nájera, 2010

Synonyms: None.

Holotype: Deposited in the Museo de Zoología de la Universidad de Costa Rica, San José, Costa Rica, 10°02'58"N, 83°32'31"W, 400–500 m.

Type locality: COSTA RICA, Limón, Guayacán de Siquirres.

Language of species description: English.

Remark: Requires revision.

70. *Peripatus swainsonae* Cockerell, 1893

Synonyms: *Peripatus jamaicensis* mut. *swainsonae*, by original designation (Cockerell 1893: 341); *Peripatus juliformis* var. *gossei* (Bouvier 1900c: 751); *Peripatus juliformis gossei* (Cockerell 1901: 326; see Remarks); *Peripatus juliformis* var. *swainsonae* (Cockerell 1901: 326; see Remarks); *Peripatus swainsonae* (Clark 1913b: 17).

Holotype: Not designated.

Type locality: JAMAICA, Saint Thomas Parish, Bath, Beacon Hill (see Remarks).

Language of species description: English.

Remarks: The name *Peripatus juliformis* var. *gossei* (Bouvier 1900c: 751) was changed to *Peripatus juliformis* var. swainsonae by Cockerell (1901: 326) due to nomenclatural and taxonomical inconsistencies. The precise locality data were obtained from labels of specimens deposited in the Natural History Museum of London, United Kingdom. Requires revision.

***Nomina dubia***

*Peripatus antiguensis* Bouvier, 1899c

Synonyms: *Peripatus antiguensis*, by original designation (Bouvier 1899c: 1345); *Peripatus dominicae* var. *antiguensis* (Bouvier 1905: 263); *Peripatus antiguensis* (Clark 1913b: 17).

Holotype: Not designated (see Remarks).

Type locality: ANTIGUA ISLAND, Barlar, near Warburton (see Remarks).

Language of species description: French.

Remarks: The holotype has not been designated explicitly in the original description. According to Bouvier (1907a: 519), a type has been deposited in the Museum National d’Histoire Naturelle de Paris, France. The current name and position of the type locality could not be found. Since no further work with more precise locality data is available, a revision of this species based on topotypes will be difficult.

*Peripatus bavaysi* Bouvier, 1899c

Synonyms: *Peripatus sedgwicki* var. *bavaysi*, by original designation (Bouvier 1899c: 1346); *Peripatus bavayi* (Clark 1913b: 17; Peck 1975: 348; see Remarks).

Holotype: Not designated (see Remarks).

Type locality: GUADELOUPE ISLAND (see Remarks).

Language of species description: French.

Remarks: Common misspelling *bavayi* (e.g., Bouvier 1899d: 415; Clark 1913b: 17; Peck 1975: 348). The holotype has not been designated explicitly in the original description. According to Bouvier (1907a: 519), a type has been deposited in the Museum National d’Histoire Naturelle de Paris, France. The description contains imprecise type locality data (the Guadeloupe Island occupies an area of 1,628 km^2^). Since no further work with more precise locality data is available, a revision of this species based on topotypes will be difficult.

##### *Plicatoperipatus* (Clark, 1913b)

Type species: *Plicatoperipatus jamaicensis* (Grabham & Cockerell, 1892), by monotypy.

71. *Plicatoperipatus jamaicensis* (Grabham & Cockerell, 1892)

Synonyms: *Peripatus jamaicensis*, by original designation (Grabham and Cockerell 1892: 514); *Peripatus jamaicensis* mut. *swainsonae* (Cockerell 1893: 341; see Remarks); *Peripatus jamaicensis* mut. *bouvieri* (Cockerell 1901: 326); *Peripatus jamaicensis* mut. *gossei* (Cockerell 1893: 341; see Remarks); *Peripatus (Plicatoperipatus) jamaicensis* (Clark 1913b: 17; see Remarks); *Plicatoperipatus jamaicensis* (Peck 1975: 349).

Holotype: Not designated (see Remarks)

Type locality: JAMAICA, Saint Thomas Parish, Bath, Beacon Hill (see Remarks).

Language of species description: English.

Remarks: The type locality data were obtained from labels of specimens deposited in the Natural History Museum of London, United Kingdom. Bouvier (1905: 171) refers to three type specimens, the location of which is uncertain. Taxonomic inconsistencies have lead Cockerell (1901: 326) to change the name *Peripatus jamaicensis* mut. *swainsonae* to *Peripatus jamaicensis* mut. *bouvieri*, but the latter may be confused with the valid species name *Peripatus bouvieri* Fuhrmann, 1913. The same holds true for *Peripatus jamaicensis* mut. *gossei*, which may be confused with *Peripatus juliformis* var. *gossei* (a synonym of *Peripatus swainsonae* Cockerell, 1893). Since the two mutations of *Plicatoperipatus* (mut. *bouvieri* and mut. *gossei*)were based only on colour variation (Cockerell 1893: 341), which in our view is insufficient, and since both mutations occur in the same area, we regard them as variations of *Plicatoperipatus jamaicensis*. A thorough revision of this species is required.

##### *Speleoperipatus* Peck, 1975

Type species: *Speleoperipatus spelaeus* Peck, 1975, by monotypy.

72. *Speleoperipatus spelaeus* Peck, 1975

Synonyms: None.

Holotype: Deposited in the Museum of Comparative Zoology at Harvard University, Cambridge, USA.

Type locality: JAMAICA, Clarendon, Pedro River, Pedro Great Cave, 518 m (1,700 ft).

Language of species description: English.

##### *Typhloperipatus* Kemp, 1913

Type species: *Typhloperipatus williamsoni* Kemp, 1913, by subsequent monotypy.

73. *Typhloperipatus williamsoni* Kemp, 1913

Synonyms: None.

Holotype: Not designated.

Type locality: INDIA, Assam, Dihang River, vicinity of Rotung, 366–762 m (1,200–2,500 ft).

Language of species description: English.

Remarks: Although the species name was introduced in 1913, a more comprehensive species description followed one year later (Kemp 1914). Requires revision

#### II. PERIPATOPSIDAE Bouvier 1905

Type genus: *Peripatopsis* Pocock, 1894

Remark:The citation “Peripatopsidae Bouvier, 1904” (e.g., Clark 1913b; Reid 1996) is inappropriate as Bouvier (1904c: 45, footnote) did not use the correct spelling Peripatopsidae but rather the French spelling “Péripatopsidés”. The name Peripatopsidae was first introduced in 1905 (Bouvier 1905: 65). It has been cited incorrectly as “Peripatopsidae Bouvier, 1907” (e.g., Ruhberg 1985), referring to Bouvier’s monograph on this taxon (Bouvier 1907b).

##### *Acanthokara* Reid, 1996

Type species: *Acanthokara kaputensis* Reid, 1996, by monotypy.

1. *Acanthokara kaputensis* Reid, 1996

Synonyms: None.

Holotype:Deposited in the Australian Museum, Sydney, Australia.

Type locality: AUSTRALIA, New South Wales, Nadewar Range, Mount Kaputar, 30°16'S, 150°10'E, 1,508 m.

Language of species description: English.

##### *Aethrikos* Reid, 1996

Type species: *Aethrikos setosa* Reid, 1996, by monotypy.

2. *Aethrikos setosa* Reid, 1996

Synonyms: None.

Holotype: Deposited in the Australian Museum, Sydney, Australia.

Type locality: AUSTRALIA, New South Wales, Styx River State Forest, 30°31'S, 152°21'E, 1,376 m.

Language of species description: English.

##### *Aktinothele* Reid, 1996

Type species: *Aktinothele eucharis* Reid, 1996, by monotypy.

3. *Aktinothele eucharis* Reid, 1996

Synonyms: None

Holotype: Deposited in the Australian National Insect Collection, Canberra, Australia.

Type locality: AUSTRALIA, Queensland, Finch Hatton Gorge, 21°05'S, 14°38'E, 200 m.

Language of species description: English.

##### *Anoplokaros* Reid, 1996

Type species: *Anoplokaros keerensis* Reid, 1996, by monotypy.

4. *Anoplokaros keerensis* Reid, 1996

Synonyms: None.

Holotype: Deposited in the Australian Museum, Sydney, Australia.

Type locality: AUSTRALIA, New South Wales, Mount Keira (near Scout Camp), 34°24'S, 150°50'E, 320 m.

Language of species description: English.

##### *Austroperipatus* Baehr, 1977

Type species: *Austroperipatus paradoxus* (Bouvier, 1914a), by subsequent monotypy.

5. *Austroperipatus aequabilis* Reid, 1996

Synonyms: None.

Holotype: Deposited in the Queensland Museum, Brisbane, Australia.

Type locality: AUSTRALIA, Queensland, Mount Finnigan, 37 km south of Cooktown, 15°49'S, 145°17'E, 850–1,100 m.

Language of species description: English.

6. *Austroperipatus eridelos* Reid, 1996

Synonyms: None.

Holotype: Deposited in the Queensland Museum, Brisbane, Australia.

Type locality: AUSTRALIA, Queensland, Boonjie, 13 km east-southeast of Malanda, 17°24'S, 145°44'E, 700 m.

Language of species description: English.

7. *Austroperipatus paradoxus* (Bouvier, 1914a)

Synonyms: *Ooperipatus paradoxus*, by original designation (Bouvier 1914a: 1548); *Austroperipatus paradoxus* (Baehr 1977: 17).

Holotype: Not designated. A lectotype has been designated by Ruhberg (1985: 115) and deposited in the Mjöberg collection at the Naturhistoriska Riksmuseet, Stockholm, Sweden (see Remarks).

Type locality: AUSTRALIA, Queensland, Bellenden Ker, 17°12'S, 145°51'E, 1,312 m (4,000 ft).

Language of species description: French

Remarks: A wrong year of description, 1915, has been assigned to the species name (e.g. Baehr 1977: 9). The holotype has not been designated in the original description. Ruhberg (1985: 115) refers to a holotype and to a paratype, which, however, should be regarded as a lectotype and as a paralectotype, respectively, as suggested by the ICZN (Art. 74).

8. *Austroperipatus superbus* Reid, 1996

Synonyms: None.

Holotype: Deposited in the Queensland Museum, Brisbane, Australia.

Type locality: AUSTRALIA, Queensland, Hinchinbrook Island, Gayundah Creek, 18°22'S, 146°13'E, 80 m.

Language of species description: English.

##### *Baeothele* Reid, 1996

Type species: *Baeothele saukros* Reid, 1996, by monotypy.

9. *Baeothele saukros* Reid, 1996

Synonyms: None.

Holotype: Deposited in the Australian Museum, Sydney, Australia.

Type locality: AUSTRALIA, New South Wales, Wollemi National Park, Mount Coricudgy, 32°50'S, 150°21'E, 1,350 m.

Language of species description: English.

##### *Centorumis* Reid, 1996

Type species: *Centorumis trigona* Reid, 1996, by monotypy.

10. *Centorumis trigona* Reid, 1996

Synonyms: None.

Holotype: Deposited in the Australian Museum, Sydney, Australia.

Type locality: AUSTRALIA, New South Wales, Gloucester Tops, 32°03'S, 151°39'E, 704 m.

Language of species description: English.

##### *Cephalofovea* Ruhberg, Tait, Briscoe and Storch, 1988

Type species: *Cephalofovea tomahmontis* Ruhberg, Tait, Briscoe and Storch, 1988, by monotypy

11. *Cephalofovea cameroni* Reid, Tait, Briscoe & Rowell, 1995

Synonyms: None.

Holotype: Deposited in the Australian Museum, Sydney, Australia.

Type locality: AUSTRALIA, New South Wales, Rydal, 33°29’S, 150°02’E, 900 m.

Language of species description: English.

12. *Cephalofovea clandestina* Reid, Tait, Briscoe & Rowell, 1995

Synonyms: None.

Holotype: Deposited in the Australian Museum, Sydney, Australia.

Type locality: AUSTRALIA, New South Wales, Kanangra Boyd National Park, 33°50'S, 150°00'E, 1,140 m.

Language of species description: English.

13. *Cephalofovea pavimenta* Reid, Tait, Briscoe & Rowell, 1995

Synonyms: None.

Holotype: Deposited in the Australian Museum, Sydney, Australia.

Type locality: AUSTRALIA, New South Wales, Mount Canobolas, 33°21'S, 148°59'E, 1,395 m.

Language of species description: English.

14. *Cephalofovea tomahmontis* Ruhberg, Tait, Briscoe & Storch, 1988

Synonyms: None.

Holotype: Deposited in the Australian National Insect Collection, Canberra, Australia.

Type locality: AUSTRALIA, New South Wales, Mount Tomah, 33°33'S, 150°25'E, 1,015 m.

Language of species description: English.

##### *Critolaus* Reid, 1996

Type species: *Critolaus lepidus* Reid, 1996, by monotypy.

15. *Critolaus lepidus* Reid, 1996

Synonyms: None.

Holotype: Deposited in the Queensland Museum, Brisbane, Australia.

Type locality: AUSTRALIA, Queensland, Calliope Range, Kroombit Tops (south-southwest of Calliope Beauty Spot 98), 24°22'S, 150°59'E, 860 m.

Language of species description: English.

##### *Dactylothele* Reid, 1996

Type species: *Dactylothele habros* Reid, 1996, by monotypy.

16. *Dactylothele habros* Reid, 1996

Synonyms: None.

Holotype: Deposited in the Queensland Museum, Brisbane, Australia.

Type locality: AUSTRALIA, New South Wales, Nothofagus Mountain, 12 km north of Woodenbong, 28°17'S, 152°38'E, 1,200 m.

Language of species description: English.

##### *Dystactotylos* Reid, 1996

Type species: *Dystactotylos aletes* Reid, 1996, by monotypy.

17. *Dystactotylos aletes* Reid, 1996

Synonyms: None.

Holotype: Deposited in the Queensland Museum, Brisbane, Australia.

Type locality: AUSTRALIA, Massey Range, 4 km west of Centre Bellenden Ker, 17°16'S, 145°49'E, 1,250 m.

Language of species description: English.

##### *Euperipatoides* Ruhberg, 1985

Type species: *Euperipatoides leuckartii* (Sänger, 1871), by monotypy.

18. *Euperipatoides kanangrensis* Reid, 1996

Synonyms: None.

Holotype: Deposited in the Australian Museum, Sydney, Australia.

Type locality: AUSTRALIA, New South Wales, Kanangra Boyd National Park, 33°59'S, 150°08'E, 1,140 m.

Language of species description: English.

19. *Euperipatoides leuckartii* (Sänger, 1871)

Synonyms: *Peripatus leuckartii*, by original designation (Sänger 1871: 257; 1900: 31; see Remarks); *Peripatus leuckarti* var. *orientalis* (Fletcher 1895: 185); *Peripatus orientalis* (Bouvier 1902d: 110); *Peripatoides leuckarti* (Bouvier 1907b: 226); *Ooperipatus leuckarti* (Baehr 1977: 13); *Euperipatoides leuckartii* (Ruhberg 1985:118).

Holotype: Not designated. A neotype has been designated by Reid (1996: 772) and deposited in the Australian Museum, Sydney, Australia (see Remarks).

Type locality:AUSTRALIA, New South Wales, Mount Tomah, 33°33'S, 150°25'E, 1,015 m.

Language of species description: Russian. French translation available (Sänger 1900; see Remarks).

Remarks: See Ruhberg (1985: 119) for synonymisation. A wrong year of description, 1869, has been commonly assigned to the species name (e.g. Ruhberg 1985; Reid 1996). However, 1869 was the year of the conference, whereas the second volume of the proceedings containing the species description was first published in 1871 (Sänger 1871). The species name is commonly misspelt as *leuckarti* (e.g., Fletcher 1888: 892; Ruhberg 1985: 118). A type was originally deposited in the Zoological collection of the Institute of Biology at the University of Leipzig, Germany, but it was presumably lost while parts of the collection were transferred to the Staatliches Museum für Tierkunde in Dresden, Germany. See Reid (1996: 774) for further information on the designation of a neotype. Note that the synonym *Peripatus leuckarti* var. *orientalis* may cause confusion with *Peripatus leuckarti* var. *occidentalis*,which is a synonym of *Kumbadjena occidentalis*.

20. *Euperipatoides rowelli* Reid, 1996

Synonyms: None.

Holotype: Deposited in the Australian Museum, Sydney, Australia.

Type locality: AUSTRALIA, New South Wales, Tallaganda State Forest, Forbes Creek Road, 35°28'S, 149°32'E, 1,000 m.

Language of species description: English.

##### *Florelliceps* Tait & Norman, 2001

Type species: *Florelliceps stutchburyae* Tait & Norman, 2001, by monotypy.

21. *Florelliceps stutchburyae* Tait & Norman, 2001

Synonyms: None.

Holotype: Deposited in the Australian National Insect Collection, Canberra, Australia.

Type locality: AUSTRALIA, New South Wales, Mount Warning National Park, 28°24'S, 153°16'E, 400 m.

Language of species description: English.

##### *Hylonomoipos* Reid, 1996

Type species: *Hylonomoipos akares* Reid, 1996, by original designation (Reid 1996: 778).

22. *Hylonomoipos akares* Reid, 1996

Synonyms: None.

Holotype: Deposited in the Australian National Insect Collection, Canberra, Australia.

Type locality: AUSTRALIA, Queensland, Lamington National Park (O’Reillys), 28°14'S, 153°08'E, 913 m.

Language of species description: English.

23. *Hylonomoipos brookensis* Reid, 1996

Synonyms: None.

Holotype: Deposited in the Queensland Museum, Brisbane, Australia.

Type locality: AUSTRALIA, Queensland, Upper Brookfield, 27°30'S, 152°55'E, 40 m.

Language of species description: English.

##### *Konothele* Reid, 1996

Type species: *Konothele kallimos* Reid, 1996, by monotypy.

24. *Konothele kallimos* Reid, 1996

Synonyms: None.

Holotype: Deposited in the Queensland Museum, Brisbane, Australia.

Type locality: AUSTRALIA, Queensland, Mount Hemmant, 6 km southwest of Cape Tribulation, 16°07'S, 145°25'E, 880 m.

Language of species description: English.

##### *Kumbadjena* Reid, 2002

Type species: *Kumbadjena occidentalis* (Fletcher, 1895), by original designation (Reid 2002: 131).

25. *Kumbadjena kaata* Reid, 2002

Synonyms: None.

Holotype: Deposited in the Western Australian Museum, Perth, Australia.

Type locality: AUSTRALIA, Western Australia, Porongurup National Park, Scenic Drive, 3.1 km west of intersection of Scenic Drive and Bolganup Road, 34°39'S, 117°51'E, 320 m.

Language of species description: English.

26. *Kumbadjena occidentalis* (Fletcher, 1895)

Synonyms: *Peripatus leuckarti* var. *occidentalis*, by original designation (Fletcher 1895: 186); *Peripatoides occidentalis* (Dakin 1920: 367); *Occiperipatoides occidentalis* (Ruhberg 1985: 126).

Holotype: Not designated. A neotype has been designated by Reid (2002: 132) and deposited in the Western Australian Museum, Perth, Australia (see Remarks).

Type locality: AUSTRALIA, Western Australia, Bridgetown Jarrah Park, 20.3 km west of intersection of South Western Highway and Brockman Highway, 34°01'S, 116°00'E, 250 m.

Language of species description: English.

Remarks: See Reid (2002: 136) for further information on the designation of a neotype. Note that the synonym *Peripatus leuckarti* var. *occidentalis* may cause confusion with *Peripatus leuckarti* var. *orientalis*,which is a synonym of *Euperipatoides leuckartii*.

27. *Kumbadjena shannonensis* Reid, 2002

Synonyms: None.

Holotype: Deposited in the Western Australian Museum, Perth, Australia.

Type locality: AUSTRALIA, Western Australia, Shannon National Park, Giant Karri Grove, Deeside Coast Road, 5 km south of intersection of Middleton Road and Deeside Coast Road, 34°38'S, 116°20'E, 150 m.

Language of species description: English.

##### *Lathropatus* Reid, 2000a

Type species: *Lathropatus nemorum* Reid, 2000a, by monotypy.

28. *Lathropatus nemorum* Reid, 2000a

Synonyms: None.

Holotype: Deposited in the Museum Victoria, Melbourne, Australia.

Type locality: AUSTRALIA, Victoria, Cobboboonee State Forest (Southern end), ~11.4 km northwest of Portland, beside Elbow Road, off Nelson Portland Road, 38°17'S, 141°33'E, 60 m.

Language of species description: English.

##### *Leuropezos* Reid, 1996

Type species: *Leuropezos eungellensis* Reid, 1996, by monotypy.

29. *Leuropezos eungellensis* Reid, 1996

Synonyms: None.

Holotype: Deposited in the Australian National Insect Collection, Canberra, Australia.

Type locality: AUSTRALIA, Queensland, Eungella National Park, Crediton Creek, 21°11'S, 148°33'E, 750 m.

Language of species description: English.

##### *Mantonipatus* Ruhberg, 1985

Type species: *Mantonipatus persiculus* Ruhberg, 1985, by monotypy.

30. *Mantonipatus persiculus* Ruhberg, 1985

Synonyms: None.

Holotype: Deposited at the Zoologisches Institut und Zoologisches Museum, University of Hamburg, Hamburg, Germany.

Type locality: AUSTRALIA, South Australia, Mount Lofty Range, Carey Gully, Wotton’s Scrub, 34°58'S, 138°46'E, 480 m.

Language of species description: German.

##### *Metaperipatus* Clark, 1913b

Type species: *Metaperipatus blainvillei* (Gervais, 1837), by monotypy.

31. *Metaperipatus inae* Mayer, 2007

Synonyms: None.

Holotype: Deposited in the Museo Zoológico de la Universidad de Concepción, Concepción, Chile.

Type locality: CHILE, VIII region del Bío-Bío, forest near Contulmo, 38°01'S, 73°11'W, 390 m.

Language of species description: English.

***Nomen dubium***

*Metaperipatus blainvillei* (Gervais, 1837)

Synonyms: *Venilia blainvillei* (Gervais 1837: 38; see Remarks); *Peripatus chiliensis* (Sedgwick 1888: 480; Wheeler 1898: 4–5); *Peripatus blainvillei* (Blanchard 1847: 140); *Peripatoides blainvillei* (Bouvier 1901b: 59); *Peripatopsis blainvillei* (Bouvier 1901b: 61); *Metaperipatus blainvillei* (Clark 1913b: 18).

Holotype: Not designated.

Type locality: CHILE (see Remarks).

Language of species description: French.

Remarks: An incorrect authority (Blanchard) is commonly attributed to this species (e.g., Clark 1913b: 18). According to Gervais (1837: 38), the species was named provisionally *Venilia blainvillei* in a letter of M. Gay to M. de Blainville. The species name is misspelt as *T. Blainvillii* Blanch. [sic] in Schmarda (1878: 77). The precise locality of the first record is unknown and the type material has been lost (Bouvier 1901b: 59). Subsequent authors recorded putative specimens of this species from a large area (e.g., Bouvier 1901b: 59; Johow 1911: 81–82; Ruhberg 1985: 108; Mayer 2007: 22). The putatively wide distribution of the species, together with an unusual variation in the number of leg pairs in specimens from different localities (Mayer 2007), indicate that *Metaperipatus blainvillei* is a species complex, which requires revision. However, the imprecise locality data and the lack of type specimens will make a revision of this species difficult.

##### *Minyplanetes* Reid, 1996

Type species: *Minyplanetes kroombensis* Reid, 1996, by monotypy.

32. *Minyplanetes kroombensis* Reid, 1996

Synonyms: None.

Holotype: Deposited in the Queensland Museum, Brisbane, Australia.

Type locality: AUSTRALIA, Queensland Kroombit Tops, 24°25'S, 151°03'E, 940 m.

Language of species description: English.

##### *Nodocapitus* Reid, 1996

Type species: *Nodocapitus barryi* Reid, 1996, by original designation (Reid 1996: 802).

33. *Nodocapitus barryi* Reid, 1996

Synonyms: None.

Holotype: Deposited in the Australian Museum, Sydney, Australia.

Type locality: AUSTRALIA, New South Wales, Richmond Range State Forest, 28°40'S, 152°45'E, 400 m.

Language of species description: English.

34. *Nodocapitus formosus* Reid, 1996

Synonyms: None.

Holotype: Deposited in the Australian National Insect Collection, Canberra, Australia.

Type locality: AUSTRALIA, Queensland, Mount Elliot, 19°29'S, 146°59'E, 1,050 m.

Language of species description: English.

35. *Nodocapitus inornatus* Reid, 1996

Synonyms: None.

Holotype: Deposited in the Australian Museum, Sydney, Australia.

Type locality: AUSTRALIA, New South Wales, Gibralter Range National Park, 29°28'S, 152°21'E, 900 m.

Language of species description: English.

##### *Occiperipatoides* Ruhberg, 1985

Type species: *Occiperipatoides gilesii* (Spencer, 1909), by original designation (Ruhberg 1985: 124; see Remarks).

Remarks: The genus originally contained two nominal species, but *Occiperipatoides occidentalis* (Fletcher, 1895) is currently treated as a synonym of *Kumbadjena occidentalis* (Fletcher, 1895) (see Ruhberg 1985; Reid 1996).

36. *Occiperipatoides gilesii* (Spencer, 1909)

Synonyms: *Peripatoides woodwardi* (junior synonym; Bouvier, 1909: 315; see Remarks); *Peripatoides gilesii* (Spencer 1909: 240); *Occiperipatoides gilesi* (Ruhberg 1985: 124).

Holotype: Not designated (Ruhberg 1985: 125; Reid 1996: 815).

Type locality: AUSTRALIA, Western Australia, Armadale, 32°09'S, 116°00'E.

Language of species description: English.

Remarks: The name *Peripatoides woodwardi* is regarded as a junior synonym since it was suggested in December 1909, while *Peripatus gilesii* was suggested in March of the same year. Thus, the name *Peripatoides woodwardi* represents a *nomen nudum*. The holotype was not clearly designated in the original description of *Occiperipatoides gilesii* (see Spencer 1909). However, Weidner (1959: 93) refers to a holotype and to a paratypoid found in the Zoologisches Staatsinstitut und Zoologisches Museum Hamburg, Germany, under the name *Peripatoides woodwardi*. Röhlig et al. (2010: 227) also refer to a paratype with the same name placed in the Museum für Naturkunde Berlin, Germany. However, these specimens cannot be regarded as types since *Peripatoides woodwardi* is a junior synonym of *Occiperipatoides gilesii*. The species name is commonly misspelt as *gilesi* (e.g., Ruhberg 1985: 124).

##### *Ooperipatellus* Ruhberg, 1985

Type species: *Ooperipatellus insignis* (Dendy, 1890), by original designation (Ruhberg 1985: 127).

37. *Ooperipatellus decoratus* (Baehr, 1977)

Synonyms: *Ooperipatus decoratus*, by original designation (Baehr 1977: 14); *Ooperipatellus insignis* (Ruhberg 1985: 128); *Ooperipatellus decoratus* (Reid 1996: 909).

Holotype: Deposited in the Australian National Insect Collection, Canberra, Australia.

Type locality: AUSTRALIA, Tasmania, Mawbanna, Dip River Falls, 8 km south of Mawbama, northwest Tasmania, ca. 250 m.

Language of species description: German.

Remarks: The species has been synonymised with *Ooperipatellus insignis* (Ruhberg 1985: 128). We regard *Ooperipatellus decoratus* and *Ooperipatellus insignis* as separate species due to the great distance (411 km) between their type localities, separated by Bass Strait. Requires revision.

38. *Ooperipatellus duwilensis* Reid, 1996

Synonyms: None.

Holotype: Deposited in the Museum Victoria, Melbourne, Australia.

Type locality: AUSTRALIA, Victoria, Grampians National Park, Mount William, 37°18'S, 142°36'E, 1,256 m.

Language of species description: English.

39. *Ooperipatellus insignis* (Dendy, 1890)

Synonyms: *Peripatus insignis*, by original designation (Dendy 1890: 174); *Ooperipatus insignis* (Dendy 1900a: 510); *Ooperipatellus insignis* (Ruhberg 1985: 127).

Holotype: Not designated.

Type locality: AUSTRALIA, Victoria, Mount Macedon, 37°23'S, 144°35'E, 1,001 m.

Language of species description: English.

Remark: The species has been revised by Reid (1996) but a lectotype still has to be designated.

40. *Ooperipatellus nanus* Ruhberg, 1985

Synonyms: None.

Holotype: Not designated.

Type locality: NEW ZEALAND, South Island, Takitimu Range, Cheviot Face, 1,160 m.

Language of species description: German.

Remarks: The species description is based on four juveniles (Ruhberg 1985: 131). Requires revision.

41. *Ooperipatellus parvus* Reid, 1996

Synonyms: None.

Holotype: Deposited in the South Australian Museum, Adelaide, Australia.

Type locality: AUSTRALIA, South Australia, Mount Lofty Range, Mylor, beside Onkaparinga river, 35°03'S, 138°46'E, 320 m.

Language of species description: English.

42. *Ooperipatellus spenceri* (Cockerell, 1913b)

Synonyms: *Ooperipatus insignis* (Bouvier 1907b: 267; see Remarks); *Ooperipatus spenceri*, by original designation (Cockerell 1913b in Clark 1913b: 19; see Remarks); *Ooperipatellus insignis* (Ruhberg 1985: 128; see Remarks).

Holotype: Not designated.

Type locality: AUSTRALIA, Tasmania, Wellington Park, Mount Wellington.

Language of species description: English.

Remarks: The species was assigned previously to *Ooperipatellus insignis* (Bouvier 1907b: 267). However, the latter species does not occur in Tasmania, which is why Cockerell (1913b; in Clark 1913b) suggested the new name *spenceri* in a footnote of Clark’s work (1913b: 19). Subsequently, the name *Ooperipatellus spenceri* was synonymised with *Ooperipatellus insignis* by Baehr (1977: 13) and Ruhberg (1985: 128). We regard *Ooperipatellus spenceri* and *Ooperipatellus insignis* as separate species, as suggested by Cockerell (1913b; in Clark 1913b), due to the great distance (652 km) between their type localities, separated by Bass Strait. Requires revision.

43. *Ooperipatellus viridimaculatus* (Dendy, 1900b)

Synonyms: *Peripatus viridimaculatus*, by original designation (Dendy 1900b: 444); *Ooperipatus viridimaculatus* (Dendy 1900a: 510); *Ooperipatellus insignis* (Ruhberg 1985: 128; see Remarks).

Holotype: Not designated.

Type locality: NEW ZEALAND, South Island, “in the dense beech forest at the head of Lake Te Anau”.

Language of species description: English.

Remarks: *Ooperipatellus viridimaculatus* was synonymised with *Ooperipatellus insignis* (Ruhberg 1985: 128). However, molecular studies indicate that specimens from New Zealand are unlikely to be conspecific with those from the type locality of *Ooperipatellus insignis* in Australia (Tait and Briscoe 1995). We regard *Ooperipatellus viridimaculatus* and *Ooperipatellus insignis* as separate species due to the great distance between their type localities, which are separated by the Tasman Sea (2,113 km between the two type localities in New Zealand and Australia). Requires revision.

***Nomen dubium***

*Ooperipatellus cryptus* Jackson & Taylor, 1994

Synonyms: None.

Holotype: Not designated.

Type locality: AUSTRALIA, Tasmania, northwest (see Remarks).

Language of species description: English.

Remarks: Imprecise type locality data have been provided for this species, covering an area of over 2,000 km^2^ in north-western Tasmania, although the “main population” occurs in the Christmas Hills, Arthur River, Rapid River area (Jackson and Taylor 1994: 167).Although the cover date for the species description is 1994, the work was not published until early 1995 (R. Mesibov *in litt*.). According to the provisions of the ICZN for publications after 1985 and before 2000, this does not constitute a published work for purposes of zoological nomenclature since it does not contain a statement that “the new name is intended for permanent scientific record” (ICZN Art. 8.5.2). Therefore, the name *Ooperipatellus cryptus* is a *nomen dubium*, which requires revision.

##### *Ooperipatus* Dendy, 1900a

Type species: *Ooperipatus oviparus* (Dendy, 1895), by subsequent designation (Dendy 1902: 367).

44. *Ooperipatus birrgus* Reid, 2000a

Synonyms: None.

Holotype: Deposited in the Museum Victoria, Melbourne, Australia.

Type locality: AUSTRALIA, New South Wales, South East Forest National Park, Coolangubra Section, 5 km north of intersection of Coolangubra Forest Way and Northern Access Road, 37°01'S, 149°23'E, 800 m.

Language of species description: English.

45. *Ooperipatus caesius* Reid, 2000a

Synonyms: None.

Holotype: Deposited in the Museum Victoria, Melbourne, Australia.

Type locality: AUSTRALIA, Victoria, Mount Buffalo National Park, Track to Eurobin Falls, 36°43'S, 146°50'E, 500 m.

Language of species description: English.

46. *Ooperipatus centunculus* Reid, 1996

Synonyms: None.

Holotype: Deposited in the Museum Victoria, Melbourne, Australia.

Type locality: AUSTRALIA, Victoria, Mount Donna Buang, 37°42'S, 145°41'E, 1,250 m.

Language of species description: English.

47. *Ooperipatus costatus* Reid, 1996

Synonyms: None.

Holotype: Deposited in the Australian Museum, Sydney, Australia.

Type locality:AUSTRALIA, Australian Capital Territory, Namadgi National Park, Stockyard Gap, 35°33'S, 148°46'E, 1,560 m.

Language of species description: English.

48. *Ooperipatus hispidus* Reid, 1996

Synonyms: None.

Holotype: Deposited in the Australian Museum, Sydney, Australia.

Type locality: AUSTRALIA, New South Wales, Tallaganda State Forest, Forbes Creek Road, 35°28'S, 149°32'E, 1,000 m.

Language of species description: English.

49. *Ooperipatus lepidus* Reid, 2000a

Synonyms: None.

Holotype: Deposited in the Museum Victoria, Melbourne, Australia.

Type locality: AUSTRALIA, Victoria, Granite Flat, 9 km south of Mitta Mitta, beside Omeo Highway, 350 m north of intersection of Omeo Highway and Walsh’s Road, 36°35'S, 147°27'E, 340 m.

Language of species description: English.

50. *Ooperipatus nebulosus* Reid, 2000a

Synonyms: None.

Holotype: Deposited in the Museum Victoria, Melbourne, Australia.

Type locality: AUSTRALIA, Victoria, Merrijig, Carters Hill Reserve, 950 m along Carters Road from Mount Buller Road, 37°06'S, 146°22'E, 640 m.

Language of species description: English.

51. *Ooperipatus oviparus* (Dendy, 1895)

Synonyms: *Peripatus oviparus*, by original designation (Dendy 1895: 195); *Ooperipatus oviparus* (Dendy 1900a: 510); *Symperipatus oviparus* (Cockerell 1913b; as a footnote in Clark 1913b: 19; see Remarks).

Holotype: Not designated. A lectotype has been deposited in the Muséum National d’Histoire Naturelle, Paris, France (Reid 1996: 830).

Type locality: AUSTRALIA, Victoria, Mount Macedon, 37°23'S, 144°35'E, 1,001 m.

Language of species description: English.

Remarks: A new genus *Symperipatus* was suggested for the species by Cockerell (1913b; as a footnote in Clark 1913b: 19). However, since *Ooperipatus oviparus* is the type species of *Ooperipatus*, the name *Symperipatus* has to be regarded as an objective synonym because it is based on the same name-bearing type (ICZN Art. 61.3.3). The species has been revised and a lectotype has been designated by Reid (1996).

52. *Ooperipatus porcatus* Reid, 2000a

Synonyms: None.

Holotype: Deposited in the Museum Victoria, Melbourne, Australia.

Type locality: AUSTRALIA, Victoria, Mount Useful Scenic Reserve, 14.5 km north of intersection of Binns Road and McEvoys Track, 37°43'S, 146°31'E, 750 m.

Language of species description: English.

53. *Ooperipatus pulchellus* Reid, 1996

Synonyms: None.

Holotype: Deposited in the Museum Victoria, Melbourne, Australia.

Type locality: AUSTRALIA, Victoria, Baw Baw National Park, Mount Baw Baw, 37°50'S, 146°17'E, 1,566 m.

Language of species description: English.

54. *Ooperipatus silvanus* Reid, 2000a

Synonyms: None.

Holotype: Deposited in the Museum Victoria, Melbourne, Australia.

Type locality: AUSTRALIA, Victoria, Otway range, 0.1 km south of intersection of Young Creek Track and Philips Road, 38°40'S, 143°30'E, 260 m.

Language of species description: English.

##### *Opisthopatus* Purcell, 1899

Type species: *Opisthopatus cinctipes* Purcell, 1899, by original designation (Purcell 1899: 349)

55. *Opisthopatus amatolensis* Choonoo, 1947

Synonyms: *Opisthopatus cinctipes* var. *amatolensis* (Choonoo 1947: 71).

Holotype: Not designated.

Type locality: SOUTH AFRICA, Eastern Cape. The precise locality might be southeast of Houghton’s farm, along the road from Alice towards Hogsback, 1,158 m (3,800 ft) (Choonoo 1947: 71).

Language of species description: English.

Remarks: The variation was considered as invalid by Ruhberg (1985: 85) and Hamer et al. (1997: 292) due to the lack of consistent differences to other subspecies. Nevertheless, we regard *Opisthopatus amatolensis* as a valid species because of the great distance between the type localities of this species and *Opisthopatus cinctipes* (161 km) and the apparent point endemism and cryptic speciation among the South African Peripatopsidae species (Daniels et al. 2009; Daniels and Ruhberg 2010; McDonald and Daniels 2012). Requires revision.

56.* Opisthopatus cinctipes* Purcell, 1899

Synonyms: None.

Holotype: Not designated.

Type locality: SOUTH AFRICA, Eastern Cape, Dunbrody, near Blue Cliff Station, Uitenhage Division.

Language of species description: English.

57. *Opisthopatus herbertorum* Ruhberg & Hamer, 2005

Synonyms: None.

Holotype: Deposited in the Natal Museum, Pietermaritzburg, South Africa.

Type locality: SOUTH AFRICA, KwaZulu-Natal, Mount Currie Nature Reserve, near Kokstad, alongside road between main entrance and pass, in forest patch near ravine, 30°17'13"S, 29°13'40"E (30.28 713°S/29.22 781°E).

Language of species description: English.

58. *Opisthopatus laevis* Lawrence, 1947

Synonyms: *Opisthopatus cinctipes* var. *laevis* by original designation (Lawrence 1947: 168).

Holotype: Not designated.

Type locality: SOUTH AFRICA, KwaZulu-Natal, East Griqualand, Bulwer.

Language of species description: English.

Remarks: The variation was considered as invalid by Ruhberg (1985: 85) due to the lack of consistent differences to other subspecies. Nevertheless, we raise *Opisthopatus laevis* to a species level and regard it as a valid species because of the great distance between the type localities of this species and *Opisthopatus cinctipes* (570 km) and the apparent point endemism and cryptic speciation among the South African Peripatopsidae species (Daniels et al. 2009; Daniels and Ruhberg 2010; McDonald and Daniels 2012). Requires revision.

59. *Opisthopatus natalensis* Bouvier, 1900d

Synonyms: *Opisthopatus cinctipes* var. *natalensis*,by original designation (Bouvier 1900d: 368).

Holotype: Not designated.

Type locality: SOUTH AFRICA, Kwa-Zulu-Natal, Durban.

Language of species description: French.

Remarks: The variation was considered as invalid by Ruhberg (1985: 85) due to the lack of consistent differences to other subspecies. Nevertheless, we raise *Opisthopatus natalensis* to a species level and regard it as a valid species because of the great distance between the type localities of this species and *Opisthopatus cinctipes* (656 km) and the apparent point endemism and cryptic speciation among the South African Peripatopsidae species (Daniels et al. 2009; Daniels and Ruhberg 2010; McDonald and Daniels 2012). Requires revision.

60. *Opisthopatus roseus* Lawrence, 1947

Synonyms: None.

Holotype: Deposited in the Natal Museum, Pietermaritzburg, South Africa.

Type locality: SOUTH AFRICA, KwaZulu-Natal, East Griqualand, Ingeli Forest, near Kokstad.

Language of species description: English.

##### *Paraperipatus* (Willey, 1898a)

Type species: *Paraperipatus novaebritanniae* (Willey, 1898b), by subsequent monotypy.

Remarks: Initially, *Paraperipatus* was described as a subgenus of *Peripatus* and the name has been used as a generic name without an explicit statement since Bouvier (1900d: 369). Most species of the genus are understudied and require thorough revisions.

61. *Paraperipatus ceramensis* (Muir & Kershaw, 1909)

Synonyms: *Peripatus ceramensis*, by original designation (Muir and Kershaw 1909: 737); *Paraperipatus ceramensis* (Horst 1910: 218).

Holotype: Not designated.

Type locality: INDONESIA, Moluccas (Maluku archipelago), West Ceram (Seram island), vicinity of Përoe.

Language of species description: English.

Remarks: Requires revision.

62. *Paraperipatus keiensis* Horst, 1923

Synonyms: None.

Holotype: Not designated.

Type locality: INDONESIA, Key Islands [Kei-islands], Great Key Island [Pulau Kai-besar], Goenoeng Daab [Gunung Daab], 300 m.

Language of species description: English.

Remarks: The species name has been misspelt as *Paraper. keyensis* in the original description (Horst 1923: 119). Requires revision.

63. *Paraperipatus novaebritanniae* (Willey, 1898b)

Synonyms: *Peripatus novæ-britanniæ*, by original designation (Willey 1898b: 286); *Peripatus (Paraperipatus) novæ-britanniæ* (Willey 1898a: 4); *Paraperipatus novæ-britanniæ* (Bouvier 1900d: 369).

Holotype: Not designated.

Type locality: PAPUA NEW GUINEA, Bismarck archipelago, New Britain island, Gazelle Peninsula, Blanche Bay, Karavi, “at an elevation of several hundred feet above see-level” (Willey 1898b).

Language of species description: English.

Remarks: Requires revision.

64. *Paraperipatus papuensis* (Sedgwick, 1910)

Synonyms: *Peripatus papuensis*, by original designation (Sedgwick 1910: 369); *Paraperipatus papuensis* (Bouvier 1914b: 222).

Holotype: Not designated.

Type locality: PAPUA NEW GUINEA, West Papua, Sarayu, Central Arfak Mountains, 1,066 m (3,500 ft).

Language of species description: English.

Remarks: Requires revision.

65. *Paraperipatus lorentzi* Horst 1910

Synonyms: none (see Remarks).

Holotype: Not designated (see Remarks)

Type locality: PAPUA NEW GUINEA, West Papua, Wichmann Mountains, southern part of the Arfak Range, 2,743 m (9,000 ft) (Brues 1921: 51).

Language of species description: English.

Remarks: A holotype has not been designated for this species, although Brues (1921: 51) refers to a female specimen as a type. The species was synonymised with *Paraperipatus papuensis* (Ruhberg 1985: 151). However, Brues (1921: 51) described morphological differences between these two species, with *Paraperipatus lorentzi* showing more similarities to *Paraperipatus stresemanni* than to *Paraperipatus papuensis* (Brues 1921: 52). Moreover, according to Brues (1921), *Paraperipatus lorentzi* and *Paraperipatus papuensis* are unlikely to have an overlapping distribution. Therefore, we regard them as separate species. Requires revision.

66. *Paraperipatus vanheurni* Horst, 1922

Synonyms: None.

Holotype: Not designated.

Type locality: PAPUA NEW GUINEA, New Guinea, Doormanpad, 2,900 m. The current name of the locality might be the Maoke Mountains (Doormanpad-bivak, Pegunungan Maoke)

Language of species description: English.

Remarks: Requires revision.

***Nomina dubia***

*Paraperipatus amboinensis* Pflugfelder, 1948

Synonyms: None.

Holotype: Not designated (see Remarks).

Type locality: INDONESIA, Maluku archipelago, Ambon Island.

Language of species description: German.

Remarks: According to Ruhberg (1985: 146), the syntypes of this species have been lost. The type locality is imprecise (Ambon Island covers an area of 775 km^2^) and a revision of the species based on topotypes will be difficult.

*Paraperipatus leopoldi* Leloup 1931

Synonyms: None (see Remarks).

Holotype: Not designated.

Type locality: PAPUA NEW GUINEA, West Papua, environs of Sakaoeni (spelt Sakoemi in the original description), 500 m.

Language of species description: French.

Remarks: In the original species description, Leloup (1931) considered *Paraperipatus lorentzi, Paraperipatus vanheurni, Paraperipatus keiensis, Paraperipatus stresemanni, Paraperipatus schultzei* and *Paraperipatus papuensis* as intermediate forms (“formes intermédiaires”) of *Paraperipatus leopoldi*. Based on the principle of priority (ICZN Art. 23), Brongersma (1932: 411) suppressed the name *leopoldi* and synonymised it with the older name *Paraperipatus papuensis*. Ruhberg (1985: 151) stated that, although the name *Paraperipatus leopoldi* is invalid, it is still available because it was accompanied by a proper species description. Accordingly, we regard *Paraperipatus leopoldi* as a *nomen dubium*. However, we disagree with Ruhberg’s (1985: 151) suggestion that *Paraperipatus leopoldi* is a junior synonym of *Paraperipatus papuensis* since the type localities of the two species lie over 145 km apart from each other.

*Paraperipatus schultzei* Heymons, 1912

Synonyms: *Paraperipatus schultzei* var. *ferrugineus* (Heymons 1912: 216; see Remarks).

Holotype: Not designated.

Type locality: PAPUA NEW GUINEA, north of New Guinea, inland region, on a mountain at 1,570 m (German New Guinea; see Remarks).

Language of species description: German.

Remarks: The holotype was not clearly designated in the original description. According to Röhlig et al. (2010: 228), the putative holotype (in fact a syntype) might have been placed in the Museum für Naturkunde Berlin but it has been lost. The more precise locality for the species might be the Sepik river system (“Sepikstrom 1,570 m”), according to Röhlig et al. (2010: 228). The putative variation *Paraperipatus schultzei* var. *ferrugineus* was found apparently in the same locality as *Paraperipatus schultzei*; this variation wassuggested based on differences in colour pattern and in the number of leg pairs (Heymons 1912: 216). These characters are known to be variable intra-specifically in the *Paraperipatus* species and wetherefore agree with Ruhberg (1985: 153) and regard *Paraperipatus schultzei* var. *ferrugineus* as synonym of *Paraperipatus schultzei*. The description of *Paraperipatus schultzei* contains imprecise locality data. Since no further work with more precise locality data is available, a revision of this species based on topotypes will be difficult.

*Paraperipatus stresemanni* Bouvier, 1914b

Synonyms: None.

Holotype: Not designated.

Type locality: INDONESIA, inland region of Ceram (Seram) Island (see Remarks).

Language of species description: French.

Remarks: Misspelt as *stresemani* by Leloup (1931). The description contains imprecise locality data (Seram occupies an area of ~17,100 km^2^). Since no further work with more precise locality data is available, a revision of this species based on topotypes will be difficult.

##### *Paropisthopatus* Ruhberg, 1985

Type species: *Paropisthopatus umbrinus* (Johow, 1911), by original designation (Ruhberg 1985: 110).

67. *Paropisthopatus umbrinus* (Johow, 1911)

Synonyms: *Peripatus (Peripatopsis) umbrinus*, by original designation (Johow 1911: 84); *Metaperipatus umbrinus* (Clark, 1915: 21; Peck, 1975: 344); *Paropisthopatus umbrinus* (Ruhberg 1985: 111).

Holotype: Not designated.

Type locality: CHILE, Valparaíso, Balneario de Zapallar, near the border of the Aconcagua province (32°33'S), La Higuera mountain, Quebrada del Tigre (300–500 m), and “point situated next to the top” (700 m).

Language of species description: Spanish.

Remarks: Requires revision.

***Nomen dubium***

*Paropisthopatus costesi* (Gravier & Fage, 1925)

Synonyms: *Opisthopatus costesi*, by original designation (Gravier and Fage 1925: 194); *Metaperipatus costesi* (Peck 1975: 344); *Paropisthopatus umbrinus* (Ruhberg 1985: 111).

Holotype: Not designated.

Type locality: CHILE, Colchagua (see Remarks).

Language of species description: French.

Remarks: The description contains imprecise type locality data (the province of Colchagua in Chile occupies 5,678 km^2^). Since no further work with more precise locality data is available, a revision of this species based on topotypes will be difficult.

##### *Peripatoides* Pocock, 1894

Type species: *Peripatoides novaezealandiae* (Hutton, 1876), by original designation (Pocock 1894: 519).

68. *Peripatoides indigo* Ruhberg, 1985

Synonyms: None.

Holotype: Deposited in the Entomology Division of the Department of Scientific and Industrial Research, Auckland, New Zealand.

Type locality: NEW ZEALAND, South Island, Nelson district, Bainham Paturau area, Twin Forks Cave, 3.22 km (2 miles) south of the Paturau river and 1.6 km (1 mile) inland from the coast.

Language of species description: German.

69. *Peripatoides kawekaensis* Trewick, 1998

Synonyms: None.

Holotype: Not designated.

Type locality: NEW ZEALAND, North Island, Hawke’s Bay, Hutchinson and Balls Clearing Reserves.

Language of species description: English.

Remarks: The original description is based exclusively on molecular data and contains no type designation, which was not mandatory until 1999 (ICZN Art. 16). However, in contrast to other species described by Trewick (1998), based on specimens from different localities, *Peripatoides kawekaensis* shows a restricted distribution. We therefore regard it as a valid species, which requires revision.

70. *Peripatoides suteri* (Dendy, 1894)

Synonyms: *Peripatus novæ-zealandiæ* var. *suteri*, by original designation (Dendy 1894: 401); *Peripatus suteri* (Dendy 1900b: 444); *Peripatoides suteri* (Bouvier 1901b: 60).

Holotype: Not designated (see Remarks).

Type locality: NEW ZEALAND, North Island, Stratford.

Language of species description: English.

Remarks: Ruhberg (1985: 142) refers to a type deposited in the Natural History Museum of London, United Kingdom, although no type specimen was designated explicitly in the original description. Requires revision.

***Nomina dubia***

*Peripatoides aurorbis* Trewick, 1998

Synonyms: None.

Holotype: Not designated (see Remarks).

Type locality: NEW ZEALAND, central and mid-northern North Island.

Language of species description: English.

Remarks: The original description is based exclusively on molecular data from specimens collected at different localities (Trewick 1998). Types were not designated and the description contains imprecise type locality data. Although molecular methods were used to define the species, no voucher specimens are available. Revision will thus be difficult.

*Peripatoides morgani* Trewick, 1998

Synonyms: None.

Holotype: Not designated.

Type locality: NEW ZEALAND, eastern North Island in a narrow coastal strip including southern and central Hawke’s Bay and north to Lake Tikitapu.

Language of species description: English.

Remarks: The original description is based exclusively on molecular data from specimens collected at different localities (Trewick 1998). Types were not designated and the description contains imprecise type locality data. Although molecular methods were used to define the species, no voucher specimens are available. Revision will thus be difficult.

*Peripatoides novaezealandiae* (Hutton, 1876)

Synonyms: *Peripatus novæ-zealandiæ*, by original designation (Hutton 1876: 361); *Peripatoides novaezealandiae* (Pocock 1894: 519).

Holotype:Not designated.

Type locality: NEW ZEALAND, southern North Island (Trewick 1998: 321). The precise locality might be near Wellington (Hutton 1876: 361; see Remarks).

Language of species description: English.

Remarks: The species name was commonly used for every species with 15 leg pairs found in New Zealand (Trewick 1998: 309). The original species description was based on specimens from different localities on both North Island and South Island. According to Trewick (1998), the species is restricted to the southern part of the North Island and the only specimens used in the original description from this area were those collected in the environs of Wellington (Hutton 1876: 361), suggesting that this might be the putative type locality of the species. However, the data available are still imprecise and different localities attributed to the species (Hutton 1876; Trewick 1998) indicate that *Peripatoides novaezealandiae* is a species complex, which requires revision.

*Peripatoides sympatrica* Trewick, 1998

Synonyms: None.

Holotype: Not designated.

Type locality: NEW ZEALAND, widespread in northern central and mid-eastern central North Island, Waitomo Caves, Saddle Road, Norsewood, Balls Clearing, Opepe, Mangatutara, Kakaho, Forthbranch and Kaueranga.

Language of species description: English.

Remarks: The original description is based exclusively on molecular data from specimens collected at different localities (Trewick 1998). Types were not designated and the description contains imprecise type locality data. Although molecular methods were used to define the species, no voucher specimens are available. Revision will thus be difficult.

##### *Peripatopsis* Pocock, 1894

Type species: *Peripatopsis capensis* (Grube, 1866), by original designation (Pocock 1894: 519).

71. *Peripatopsis alba* Lawrence, 1931

Synonyms: None.

Holotype: Likely to have been deposited in the South African Museum, Cape Town, South Africa (see Remarks).

Type locality: SOUTH AFRICA, Cape Town, Table Mountain Nature Reserve, Table Mountain Caves, in sandstone formation near the top of Table Mountain.

Language of species description: English.

Remarks: The author designated two types in the original description but did not specify, which of the two specimens is the holotype (Lawrence 1931: 104). Furthermore, Lawrence (1931) did not provide any information on deposition of specimens, but they might have been deposited in the South African Museum (Cape Town, South Africa) as the author worked at this institution while describing the species.

72. *Peripatopsis balfouri* (Sedgwick, 1885)

Synonyms: *Peripatus balfouri*, by original designation (Sedgwick 1885: 450); *Peripatopsis balfouri* (Purcell 1899: 341).

Holotype: Not designated.

Type locality: SOUTH AFRICA, Cape Town, Table Mountain Nature Reserve (see Remarks).

Language of species description: English.

Remarks: The species comprises a species complex according to Daniels et al. (2009) and we suggest that the name should be used only for specimens obtained from the type locality. Requires revision.

73. *Peripatopsis capensis* (Grube, 1866)

Synonyms: *Peripatus capensis*, by original designation (Grube 1866: 65); *Peripatopsis capensis* (Pocock 1894: 519).

Holotype: Not designated. A neotype has been designated by Ruhberg (1985: 94) and deposited in the Zoologisches Museum, Hamburg, Germany (see Remarks).

Type locality: SOUTH AFRICA, Cape Town, Table Mountain, Rhodes Memorial (see Remarks).

Language of species description: German.

Remarks: A type specimen has not been designated in the original description. Bouvier (1907b: 146) stated that the syntypes were lost and therefore a neotype has been designated by Ruhberg (1985: 94). The species was regarded as a species complex (Daniels et al. 2009) and revised using molecular and morphological methods (McDonald and Daniels 2012; McDonald et al. 2012). The name *Peripatopsis capensis* should be used only for specimens obtained from the type locality.

74. *Peripatopsis clavigera* Purcell, 1899

Synonyms: None.

Holotype: Not designated.

Type locality: SOUTH AFRICA, Western Cape, “in the forest at Knysna”. The precise locality might be the Garden of Eden (Ruhberg 1985: 96; see Remarks).

Language of species description: English.

Remarks: The species comprises a species complex according to Daniels et al. (2009). Thus, the name should be applied only to specimens obtained from the type locality. Requires revision.

75. *Peripatopsis intermedia* Hutchinson, 1928

Synonyms: None.

Holotype: Deposited in the South African Museum, Cape Town, South Africa(see Remarks).

Type locality: SOUTH AFRICA, 11.26 km (7 miles) east of Montagu.

Language of species description: English.

Remarks: A holotype has been designated in the original description under the name type. Ruhberg (1985: 91) has synonymised the species with *Peripatopsis balfouri* due to the bad condition of the type (known from the original description) and on putatively unreliable characters, on which the species was based. However, we believe it is premature to conclude that these species names are synonyms as long as no additional specimens of *Peripatopsis intermedia* have been collected and the species re-described. Moreover, the type localities of *Peripatopsis balfouri* and *Peripatopsis intermedia* lie 168 km apart. We therefore consider *Peripatopsis intermedia* as a valid species, which requires revision.

76. *Peripatopsis lawrencei* McDonald, Ruhberg & Daniels, 2012

Synonyms: *Peripatopsis capensis* (Daniels et al. 2009: 203).

Holotype: Deposited in the South African Museum – Entomological Collection (Iziko Museums of Cape Town), Cape Town, South Africa.

Type locality: SOUTH AFRICA, Western Cape province, Riviersonderend, Oubos, 34°04'34.33"S, 19°49'43.76"E (see Remarks).

Language of species description: English.

Remarks: McDonald et al. (2012) described the species based on specimens from different localities. Their phylogenetic analysis suggests disconnected haplotype networks and additional monophyletic clades within *Peripatopsis lawrencei*, which shows a wide distribution(McDonald and Daniels 2012; McDonald et al. 2012). This indicates that *Peripatopsis lawrencei* is a species complex. Thus, the name *Peripatopsis lawrencei* should be applied only to specimens obtained from the type locality.

77. *Peripatopsis leonina* Purcell, 1899

Synonyms: None.

Holotype: Not designated (see Remarks).

Type locality: SOUTH AFRICA, Cape Town, Cape town side of Signal Hill (Lions Hill).

Language of species description: English.

Remarks: A type specimen has not been designated explicitly in the original description, although Weidner (1959: 93) and Ruhberg (1985: 98) refer to paratypoids and ex-types, the taxonomic status of which is unclear. No further record of this species has been made since 1912 (NHM-1936.4.28.4), suggesting that the species is either extinct or critically endangered (Brinck 1957: 13; Ruhberg 1985: 98; Daniels et al. 2009). Revision is required but will be difficult to accomplish, given the rarity of the specimens and the critically endangered status of the species.

78. *Peripatopsis moseleyi* (Wood-Mason, 1879)

Synonyms: *Peripatus moseleyi*, by original designation (Wood-Mason 1879: 155; see Remarks); *Peripatopsis moseleyi* (Purcell 1899: 338).

Holotype: Not designated.

Type locality: SOUTH AFRICA, Eastern Cape, presumably the environs of King William’s town (Ruhberg 1985: 100; see Remarks).

Language of species description: English.

Remarks: Species name suggested in a footnote in the original paper (Wood-Mason 1879: 155). The species comprises a species complex according to Daniels et al. (2009). Thus, the species name should be applied only to specimens obtained from the type locality. Requires revision.

79. *Peripatopsis overbergiensis* McDonald, Ruhberg & Daniels, 2012

Synonyms: *Peripatopsis capensis* (Daniels et al. 2009: 203).

Holotype: Deposited in the South African Museum – Entomological Collection (Iziko Museums of Cape Town), Cape Town, South Africa.

Type locality: SOUTH AFRICA, Western Cape province, Langeberg, Grootvadersbosch Nature Reserve, 33°58'55"S, 20°49'23"E (see Remarks).

Language of species description: English.

Remarks: McDonald et al. (2012) described the species based on specimens from different localities. Their phylogenetic analysis suggests disconnected haplotype networks and additional monophyletic clades within *Peripatopsis overbergiensis*, which shows a wide distribution(McDonald and Daniels 2012; McDonald et al. 2012). This indicates that *Peripatopsis overbergiensis* is a species complex. Thus, the name *Peripatopsis overbergiensis* should be applied only to specimens obtained from the type locality.

80. *Peripatopsis sedgwicki* Purcell, 1899

Synonyms: *Peripatus dewaali* (senior synonym: Weber, 1898: 8); *Peripatopsis sedgwicki* (Purcell 1899: 345; see Remarks).

Holotype: Not designated.

Type locality: SOUTH AFRICA, Western Cape, most likely the environs of Knysna (Weber 1898: 8; see Remarks).

Language of species description: Dutch.

Remarks: Although *Peripatopsis sedgwicki* is a junior synonym of *Peripatus dewaali*, the name *sedgwicki* was favoured by Ruhberg (1985: 102) due to its putative long usage (see also the ICZN Art. 11.6.1). For the sake of stability, we consider *Peripatopsis dewaali* as a *nomen oblitum* and *Peripatopsis sedgwicki* as a *nomen protectum*, following the ICZN (Art. 23). However, since the epithet *sedgwicki* has also been assigned to *Peripatus sedgwicki*, a representative of Peripatidae, the abbreviation *Peripatopsis sedgwicki* may cause confusion between these two species (see Röhlig 2010: 230). Recently, Expressed Sequence Tags (ESTs) have become available from a putative specimen of *Peripatopsis sedgwicki* (Meusemann et al. 2010). Thus, reverting the species name back to *Peripatopsis dewaali* at this point would create additional instability. We therefore suggest that the name *Peripatopsis sedgwicki* should be used with caution, in particular when additional data become available for both *Peripatus sedgwicki* and *Peripatopsis sedgwicki*. Furthermore, the name *Peripatopsis sedgwicki* should be applied only to specimens from the type locality as recent evidence suggests that it represents a species complex (Daniels et al. 2009). Requires revision.

81. *Peripatopsis stelliporata* Sherbon & Walker, 2004

Synonyms: None.

Holotype: Deposited in the Natural History Museum of London, United Kingdom.

Type locality: SOUTH AFRICA, Cape Town, Newlands Forest, 33°12’S, 18°24’E.

Language of species description: English.

##### *Phallocephale* Reid, 1996

Type species: *Phallocephale tallagandensis* Reid, 1996, by monotypy.

82. *Phallocephale tallagandensis* Reid, 1996

Synonyms: None.

Holotype: Deposited in the Australian Museum, Sydney, Australia.

Type locality: AUSTRALIA, New South Wales, Tallaganda State Forest, Forbes Creek Road, 35°28'S, 149°32'E, 1,000 m.

Language of species description: English.

##### *Planipapillus* Reid, 1996

Type species: *Planipapillus taylori* Reid, 1996, by original designation (Reid 1996: 851).

83. *Planipapillus annae* Reid, 2000b

Synonyms: None.

Holotype: Deposited in the Museum Victoria, Melbourne, Australia.

Type locality: AUSTRALIA, Victoria, 5.9 km northwest of Bonang, beside Deddick River Road (between Bonang and Tubbut), 37°11'S, 148°41'E, 740 m.

Language of species description: English.

84. *Planipapillus berti* Reid, 2000b

Synonyms: None.

Holotype: Deposited in the Museum Victoria, Melbourne, Australia.

Type locality: AUSTRALIA, Victoria, Granite Flat, 9 km south of Mitta Mitta, beside Omeo Highway, north of intersection of Omeo Highway and Walsh’s Road, 36°35'S, 147°27'E, 350 m.

Language of species description: English.

85. *Planipapillus biacinaces* Reid, 1996

Synonyms: None.

Holotype: Deposited in the Museum Victoria, Melbourne, Australia.

Type locality: AUSTRALIA, Victoria, Howman Gap, 36°50'S, 147°16'E, 1,260 m.

Language of species description: English.

86. *Planipapillus biacinoides* Reid, 2000b

Synonyms: None.

Holotype: Deposited in the Museum Victoria, Melbourne, Australia.

Type locality: AUTRALIA, Victoria, beside Livingtone Creek at intersection of Birregun Road and Upper Livingstone Track (6.2 km south of intersection of Cassilis Road and Birregun Road), 37°05'S, 147°36'E, 300 m.

Language of species description: English.

87. *Planipapillus bulgensis* Reid, 1996

Synonyms: None.

Holotype: Deposited in the Museum Victoria, Melbourne, Australia.

Type locality: AUSTRALIA, Victoria, Tarra-Bulga National Park, 38°26'S, 146°32'E, 580 m.

Language of species description: English.

88. *Planipapillus cyclus* Reid, 2000b

Synonyms: None.

Holotype: Deposited in the Museum Victoria, Melbourne, Australia.

Type locality: AUSTRALIA, Victoria, 9 km north of Club Terrace, junction of Errinundra Road and Combienbar Road, 37°28'S, 148°55'E, 130 m.

Language of species description: English.

89. *Planipapillus gracilis* Reid, 2000b

Synonyms: None.

Holotype: Deposited in the Museum Victoria, Melbourne, Australia.

Type locality: AUSTRALIA, Victoria, beside Livingstone Creek, at intersection of Birregun Road and Upper Livingstone Track (6.2 km south of Cassilis Road and Birregun Road), 37°05'S, 147°36'E, 300 m.

Language of species description: English.

90. *Planipapillus impacris* Reid, 2000b

Synonyms: None.

Holotype: Deposited in the Museum Victoria, Melbourne, Australia.

Type locality: AUSTRALIA, New South Wales, South East Forests National Park, Coolangubra Section, 5 km north of intersection of Coolangubra Forest Way and Northern Access Road, 37°01'S, 149°23'E, 800 m.

Language of species description: English.

91. *Planipapillus mundus* Reid, 1996

Synonyms: None.

Holotype: Deposited in the Australian Museum, Sydney, Australia.

Type locality: AUSTRALIA, New South Wales, Wilsons Valley, 36°21'S, 148°32'E, 1,360 m.

Language of species description: English.

92. *Planipapillus taylori* Reid, 1996

Synonyms: None.

Holotype: Deposited in the Australian Museum, Sydney, Australia.

Type locality: AUSTRALIA, New South Wales, Bombala River, 36°37'S, 149°22'E, 1,120 m.

Language of species description: English.

93. *Planipapillus tectus* Reid, 2000b

Synonyms: None.

Holotype: Deposited in the Museum Victoria, Melbourne, Australia.

Type locality: AUSTRALIA, Victoria, 6.7 km south of the intersection of Gelantipy Road and Tulloch Ard Road (10.7 km south of Gelantipy, 300 m north of Forest Creek Track), 37°17'S, 148°15'E, 710 m.

Language of species description: English.

94. *Planipapillus vittatus* Reid, 2000b

Synonyms: None.

Holotype: Deposited in the Museum Victoria, Melbourne, Australia.

Type locality: AUSTRALIA, Victoria, Dinner Plain, 36°59’S, 147°17’E, 1,628 m.

Language of species description: English.

##### *Regimitra* Reid, 1996

Type species: *Regimitra quadricaula* Reid, 1996, by monotypy.

95. *Regimitra quadricaula* Reid, 1996

Synonyms: None.

Holotype: Deposited in the Australian Museum, Sydney, Australia.

Type locality: AUSTRALIA, New South Wales, Tuggolo State Forest, 31°31'S, 151°27'E, 1,060 m.

Language of species description: English.

##### *Ruhbergia* Reid, 1996

Type species: *Ruhbergia bifalcata* Reid, 1996, by original designation (Reid 1996: 868).

96. *Ruhbergia bifalcata* Reid, 1996

Synonyms: None.

Holotype: Deposited in the Australian Museum, Sydney, Australia.

Type locality: AUSTRALIA, New South Wales, Tinderry Mountains, 35°40'S, 149°15'E, 1,300 m.

Language of species description: English.

97. *Ruhbergia brevicorna* Reid, 1996

Synonyms: None.

Holotype: Deposited in the Australian Museum, Sydney, Australia.

Type locality: AUSTRALIA, New South Wales, Mount Fairy (northwestern Bungendore), 35°09'S, 149°33'E, 820 m.

Language of species description: English.

98. *Ruhbergia rostroides* Reid, 1996

Synonyms: None.

Holotype: Deposited in the Australian Museum, Sydney, Australia.

Type locality: AUSTRALIA, New South Wales, Wombeyan Caves, intersection of Wombeyan Caves Road and Langs Road, 34°18'S, 150°01'E, 420 m.

Language of species description: English.

##### *Sphenoparme* Reid, 1996

Type species: *Sphenoparme hobwensis* Reid, 1996, by monotypy.

99. *Sphenoparme hobwensis* Reid, 1996

Synonyms: None.

Holotype: Deposited in the Queensland Museum, Brisbane, Australia.

Type locality: AUSTRALIA, Queensland, Lamington National Park, Mount Hobwee, 28°15'S, 153°14'E, 500 m.

Language of species description: English.

##### *Tasmanipatus* Ruhberg, Mesibov, Briscoe and Tait, 1991

Type species: *Tasmanipatus barretti* Ruhberg, Mesibov, Briscoe & Tait, 1991, by original designation (Ruhberg et al. 1991: 7).

100. *Tasmanipatus anophthalmus* Ruhberg, Mesibov, Briscoe & Tait, 1991

Synonyms: None.

Holotype: Deposited in the Queen Victoria Museum and Art Gallery, Launceston, Australia.

Type locality: AUSTRALIA, Tasmania, Elephant Pass, 4l°38'S, 148°14'E, 380 m.

Language of species description: English.

101. *Tasmanipatus barretti* Ruhberg, Mesibov, Briscoe & Tait, 1991

Synonyms: None.

Holotype: Deposited in the Queen Victoria Museum and Art Gallery, Launceston, Australia.

Type locality: AUSTRALIA, Tasmania, Evercreech Rivulet, 41°27'S, 147°57'E.

Language of species description: English.

##### *Tetrameraden* Reid, 1996

Type species: *Tetrameraden meringos* Reid, 1996, by monotypy.

102. *Tetrameraden meringos* Reid, 1996

Synonyms: None.

Holotype: Deposited in the Australian Museum, Sydney, Australia.

Type locality: AUSTRALIA, New South Wales, Warrumbungle Range, Siding Springs Mountain, 31°16'S, 149°04'E, 1,165 m.

Language of species description: English.

##### *Vescerro* Reid, 1996

Type species: *Vescerro turbinatus* Reid, 1996, by monotypy.

103. *Vescerro turbinatus* Reid, 1996

Synonyms: None.

Holotype: Deposited in the Queensland Museum, Brisbane, Australia.

Type locality: AUSTRALIA, Queensland, Iron Range, Claudie River, 12°45'S, 143°14'E, 50 m.

Language of species description: English.

##### *Wambalana* Reid, 1996

Type species: *Wambalana makrothele* Reid, 1996, by monotypy.

104. *Wambalana makrothele* Reid, 1996

Synonyms: None.

Holotype: Deposited in the Australian Museum, Sydney, Australia.

Type locality: AUSTRALIA, New South Wales, Telegherry State Forest, 32°07'S, 151°41'E, 900 m.

Language of species description: English.

#### III. FOSSIL SPECIES WITH UNCERTAIN RELATIONSHIP TO THE EXTANT TAXA

##### †*Cretoperipatus* Grimaldi,Engel & Nascimbene, 2002

Type species: †*Cretoperipatus burmiticus* Grimaldi et al. ,2002, by monotypy.

1. †*Cretozperipatus burmiticus* Grimaldi et al. ,2002

Synonyms: None.

Holotype: Deposited in the American Museum of Natural History, New York, USA.

Type locality: MYANMAR, Kachin, Tanai Village (on Ledo Road, 105 km northwest of Myitkyina).

Language of species description: English.

Remarks: Species description is based on an incomplete specimen preserved in amber of ambiguous age. Grimaldi et al. (2002) assigned the species to the Peripatidae, but this relationship is uncertain due to a limited preservation of the specimen. Note that the preserved body portion might be the posterior rather than the anterior end and the structure labelled mouth by Grimaldi et al. (2002: fig. 17) might be the genital pad.

##### †*Succinipatopsis* Poinar, 2000

Type species: †*Succinipatopsis balticus* Poinar, 2000, by monotypy.

2. †*Succinipatopsis balticus* Poinar, 2000

Synonyms: None.

Holotype: Deposited in the Poinar amber collection at the Oregon State University, Corvallis, USA.

Type locality: BALTIC REGION.

Language of species description: English.

Remarks: In amber, dating back to ~40 million years. Poinar (2000) assigned †*Succinipatopsis balticus* to a new taxon, Succinipatopsidae, the validity of which is doubtful (Grimaldi et al. 2002).

##### †*Tertiapatus* Poinar, 2000

Type species: †*Tertiapatus dominicanus* Poinar, 2000, by monotypy.

3. †*Tertiapatus dominicanus* Poinar, 2000

Synonyms: None.

Holotype: Deposited in the Poinar amber collection at Oregon State University, Corvallis, USA.

Type locality: DOMINICAN REPUBLIC.

Language of species description: English.

Remarks: In amber, dating back to 15–45 million years. Poinar (2000) assigned †*Tertiapatus dominicanus* to a new taxon, Tertiapatidae, the validity of which is doubtful (Grimaldi et al. 2002).

## Discussion

Our revised checklist revealed 177 valid onychophoran species worldwide (Table 1). However, this number most likely does not reflect the actual diversity of Onychophora as recent evidence suggests a high number of undescribed and cryptic species in both Peripatidae and Peripatopsidae (Reid 1996; Daniels et al. 2009; Lacorte 2011; Oliveira et al. 2011; McDonald and Daniels 2012; McDonald et al. 2012). Despite our attempt to retain as many valid species as possible, about 10% of described species represent *nomina dubia* (Table 1). The designation of *nomina dubia* in our list means that the species names are still available but difficult to revise due to the lack of designated types and precise locality data.

Besides the *nomina dubia*, our study shows that many of the valid species also require revision, in particular representatives of *Paraperipatus* within Peripatopsidae, and nearly all species of Peripatidae. It is evident from previous studies that numerous species have been described based on characters that are ambiguous for some onychophoran subgroups, such as colour pattern of the skin, number of leg pairs, number of jaw denticles, and number and arrangement of spinous pads (for critical discussions, see Read 1988a; Reid 1996; Oliveira et al. 2011). Typically, these species cannot be identified reliably without their locality data. Thus, a thorough revision of these species using scanning electron microscopy and molecular markers is desirable because these methods have proven useful for studies of the onychophoran taxonomy (e.g., Read 1988a, 1988b; Reid 1996; Daniels et al. 2009; Oliveira et al. 2011). The use of these methods will inevitably alter the traditional classification since several onychophoran genera, in particular within Peripatidae, are not based on unique features or synapomorphies but might be subjective, non-monophyletic assemblages (Oliveira et al. 2010, 2011).

**Table 1.** Summary of the number of species and genera within Onychophora and their taxonomic status.<br/>

	**Peripatidae**	**Peripatopsidae**	**Fossil taxa**
Number of genera	10	39	3
Number of valid species	73	104	3
Number of *nomina dubia*	9	11	–
Number of species with designated holotypes	28	81	3

Notably, 70% of the Peripatopsidae species (81 species) but only 34% of the Peripatidae (28 species) have designated holotypes (Table 1; Appendix 1: Checklist_numbers). This finding reflects the understudied nature of Peripatidae, with only one thorough revision at the species level (Bouvier 1905), while there have been at least four comprehensive revisions of the Peripatopsidae species from different geographic regions (Bouvier 1907b; Ruhberg 1985; Reid 1996; Hameret al. 1997). This highlights the urgent need of more taxonomic work on the Peripatidae species.

## Appendix

Summary of the current status of species within Onychophora. (doi: 10.3897/zookeys.211.3463.app) File format: Adobe PDF File (pdf).

**Explanation note:** Number of valid species, *nomina dubia* and designated holotypes within each onychophoran genus.
